# Metabolite and transcriptome analysis during fasting suggest a role for the p53-Ddit4 axis in major metabolic tissues

**DOI:** 10.1186/1471-2164-14-758

**Published:** 2013-11-05

**Authors:** Michael Schupp, Fang Chen, Erika R Briggs, Shilpa Rao, Helmut J Pelzmann, Ariane R Pessentheiner, Juliane G Bogner-Strauss, Mitchell A Lazar, Don Baldwin, Andreas Prokesch

**Affiliations:** 1 Institute for Genomics and Bioinformatics, Graz University of Technology, Petersgasse 14, Graz 8010, Austria; 2 Institute of Biochemistry, Graz University of Technology, Petersgasse 12/2, Graz 8010, Austria; 3 Department of Endocrinology, Diabetes, and Nutrition and Center for Cardiovascular Research (CCR), Charité University Medicine, Hessische Str. 3-4, Berlin 10115, Germany; 4 Department of Microbiology, 201 Johnson Pavilion, Perelman School of Medicine University of Pennsylvania, 3610 Hamilton Walk, Philadelphia, PA 19104, USA; 5 Pathonomics LLC, Suite 200, 3160 Chestnut St., Philadelphia, PA 19104, USA; 6 Penn Bioinformatics Core, University of Pennsylvania, Philadelphia, PA 19104, USA; 7 Department of Medicine, and the Institute for Diabetes, Obesity, and Metabolism, Division of Endocrinology, Diabetes, and Metabolism, Perelman School of Medicine at the University of Pennsylvania, Philadelphia, PA 19104, USA

**Keywords:** Fasting, Starvation, Nutrient deprivation, Adipose tissue, p53 signaling, Ddit4, Lipolysis

## Abstract

**Background:**

Fasting induces specific molecular and metabolic adaptions in most organisms. In biomedical research fasting is used in metabolic studies to synchronize nutritional states of study subjects. Because there is a lack of standardization for this procedure, we need a deeper understanding of the dynamics and the molecular mechanisms in fasting.

**Results:**

We investigated the dynamic changes of liver gene expression and serum parameters of mice at several time points during a 48 hour fasting experiment and then focused on the global gene expression changes in epididymal white adipose tissue (WAT) as well as on pathways common to WAT, liver, and skeletal muscle. This approach produced several intriguing insights: (i) rather than a sequential activation of biochemical pathways in fasted liver, as current knowledge dictates, our data indicates a concerted parallel response; (ii) this first characterization of the transcriptome signature of WAT of fasted mice reveals a remarkable activation of components of the transcription apparatus; (iii) most importantly, our bioinformatic analyses indicate p53 as central node in the regulation of fasting in major metabolic tissues; and (iv) forced expression of Ddit4, a fasting-regulated p53 target gene, is sufficient to augment lipolysis in cultured adipocytes.

**Conclusions:**

In summary, this combination of focused and global profiling approaches provides a comprehensive molecular characterization of the processes operating during fasting in mice and suggests a role for p53, and its downstream target Ddit4, as novel components in the transcriptional response to food deprivation.

## Background

Fasting processes are evolutionarily highly conserved adaptive responses to food deprivation in all organisms. Mammals follow a day/night pattern that includes a daily resting period during which the body is in a fasted state and must adapt to the lack of nutrient intake by changing the metabolic state in several organs and at the systemic level. When deprived of dietary nutrients, the body initially derives glucose from glycogen stores, which are quickly depleted [1]. If fasting continues, peripheral organs switch from glucose to fatty acids as the primary energy source. These fatty acids are mainly supplied from adipose tissue stores where they are released from triglyceride droplets by the process of lipolysis [2]. However, the brain is not equipped to derive energy from fatty acids, but uninterrupted maintenance of its function is essential to organismal survival. Hence, the perhaps most astounding metabolic change in fasting is the massive increase in the hepatic production of ketone bodies, which the brain can use as a secondary energy source [3]. The emergence of biochemical pathways that produce and then utilize ketone bodies is believed to be a major selective force in the evolutionary history of Homo sapiens and this adaptive process, along with others, enables human adults to survive for more than two months without food intake [1]. While this is crucial for survival of millions in poor, under-developed countries, Western societies are facing a pandemic of obesity with more than one third of the population being obese in countries like the United States of America [4]. In this context, fasting is still one of the most efficient weight loss measures and therefore a valuable therapeutic tool for the management of obesity [5]. Indeed, caloric restriction and alternate-day fasting have both been shown to increase longevity [6] and reduce metabolic risk factors [7]. Further, a ketogenic state as in fasting can also be elicited by low-carbohydrate diets which are among the most efficient weight loss programs [8] and are also used in clinical settings to treat forms of epilepsy [9]. For these reasons it is imperative to add to the current knowledge about the molecular underpinnings and the systemic consequences of fasting.

In experimental animals, fasting is frequently used in studies where nutritional states need to be synchronized in order to keep biological variation (introduced by varying patterns of food intake) minimal and, thereby, to provide a steady baseline for all measured parameters. The major problem with this approach is that there is no standard protocol that the research community adheres to and studies report fasting regimes in a wide range (from a few hours to days) and with different day-time starting points, often disregarding the intricate circadian regulation on the organ and the systemic level [10]. Consequently, a systematic meta-study identified fasting as one of the main sources of variation between different transcriptomic studies [11]. Hence, to estimate the impact on measured experimental parameters when animals are fasted, a deeper understanding of this process is needed. For instance, whereas the fasting response of several mouse tissues (liver, brain, gut, muscle, kidney) has been investigated at the transcriptome [12,13] and proteome [14] level, a comprehensive view on gene regulation in white adipose tissue of fasted mice is still missing.

In this work we investigated the dynamic and circadian responses to a fasting stimulus by measuring serum parameters and liver gene expression in fasted mice at several time points. Moreover, we measured the global transcriptome response to fasting in white adipose tissue, liver, and skeletal muscle utilizing microarray technology followed by a host of bioinformatic analyses. Interesting outcomes from the fasted adipose tissue data include a strong enrichment of upregulated genes coding for proteins that regulate transcription as well as apoptosis-related genes. A major finding is the identification of the p53 signaling pathway as a common mediator of the fasting response in all three investigated tissues. Driven by these results we focused on the p53-target gene Ddit4, which was upregulated by fasting in all three tissues. Our experiments showed that, in cultured adipocytes, Ddit4 is inducible by p53 activation and its ectopic expression is sufficient to augment lipolysis. Therefore we describe a new molecular component in the fasting-response downstream of p53.

## Results and discussion

### Kinetics of serum parameters and liver gene expression in mice during a 48 hour fasting period

Despite a considerable amount of knowledge about the mechanisms of fasting response in different organisms (e.g. [1,12]), its exact timing, especially at time points earlier than 12 hours, is still unclear. To assess the timely fasting response of serum metabolites we measured blood glucose, non-esterified fatty acids (NEFA), glycerol, and β-hydroxybutyrate, as well as the hormones insulin and corticosterone, at the start of the experiment and at five time points during a 48 hour period (0, 3, 6, 12, 24 and 48 hours) both in fasted and in control (i.e. ad-libitum fed) male C57Bl/6 J mice. The experiment was started at the beginning of the light cycle (9 a.m.) when mice are in their inactive phase. For these time points we determined body weight and weight changes within each group (Table 1) and measured the expression of genes in pathways central to the fasting response in liver, which is considered the main organ for maintaining systemic energy homeostasis.

**Table 1:** Weight changes of study animals

		**Absolute weight [g]**		**Weight change to initial weight within groups [g]**	
**ZT**		**Fed (n = 5)**	**Fasted (n = 5)**	**Fed (n = 5)**	**Fasted (n = 5)**
2	0 h	26.7 ± 0.6	-	−0.21 ± 0.62	-
5	3 h	28 ± 0.74	26.2 ± 0.65	−0.13 ± 0.14	−2.13 ± 0.2
8	6 h	25.5 ± 1.73	28 ± 0.68	−1.14 ± 0.11	−2.26 ± 0.19
14	12 h	26.8 ± 0.93	24.8 ± 0.57	−1.09 ± 0.15	−2.96 ± 0.14**
26	24 h	25.1 ± 0.69	22.9 ± 0.33	−0.48 ± 0.15	−3.5 ± 0.14**
50	48 h	26.9 ± 0.44	22 ± 0.55***	−0.66 ± 0.29	−5.9 ± 0.42***

Significant differences were determined with a 2-way ANOVA followed by a Bonferroni posttest to determine significance for each time point (in bold: **p < 0.01; ***p < 0.001). ZT: zeitgeber time (hours).

### Immediate upregulation of hepatic gluconeogenesis by fasting

**Figure 1:**
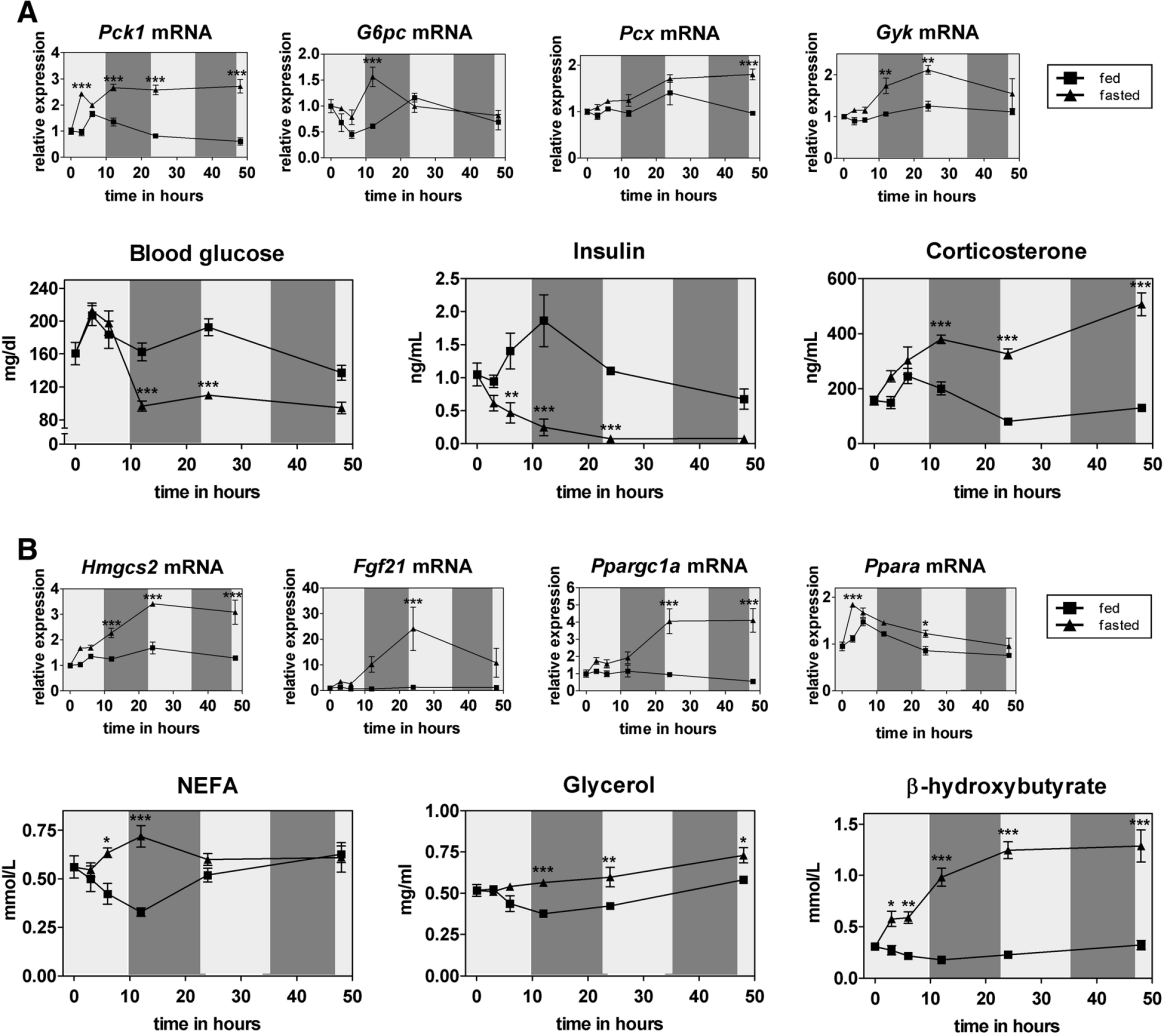
**Kinetics of selected metabolites/hormones and expression of liver genes during a 48 hour fasting period. (A)** and **(B)** Serum parameters and liver mRNA levels were measured in 10–12 weeks old fasted and ad-libitum fed male C57Bl/6 J mice at several time points (each n = 5). Shading indicates light or dark cycle. Relative expression values from qPCR measurements were normalized to 36b4 expression and the values for time point 0 were set to 1. Significance was determined with a 2-way ANOVA followed by a Bonferroni posttest to determine significance for the single time points (*p < 0.05; **p < 0.01; ***p < 0.001).

A well-known and essential transcriptional response to fasting is the upregulation of *Pck1* and *G6pc* mRNAs [15-17]. Pck1 (all gene names referring to mRNA transcripts in this article are in italic and given according to the unique NCBI gene symbol nomenclature; full names can be found in the abbreviations list) participates in controlling the flux into the gluconeogenesis (GNG) pathway, while G6pc catalyzes the last step in this pathway converting glucose-6-phosphate to glucose. Activation of liver GNG is essential in times of undernutrition to provide the brain with glucose, its primary energy source. We found that this upregulation occurs already after 3 hours fasting and continues, at least in case of *Pck1* mRNA, throughout 48 hours of fasting (Figure 1A top row). Further, we measured continuous upregulation of *Pcx* as well as *Gyk* (Figure 1A top row). Pcx converts pyruvate to oxaloacetate, which in turn serves as substrate for Pck1. Gyk, which has already been shown to be upregulated by fasting in a Ppara-dependent manner [18], catalyzes the first step in the glycerol phosphate shuttle which utilizes glycerol released from triglyceride stores to be converted into lipids or shunted into the GNG pathway. In accordance with the early upregulation of gluconeogenic genes, serum levels of corticosterone (glucocorticoids are known as positive regulators of hepatic GNG by regulating Pck1 and G6pc levels via the glucocorticoid receptor [19,20]) rose immediately after onset of fasting, showing >4-fold higher levels later in the fasting period (Figure 1A bottom row). However, during the first 6 hours of fasting, blood glucose levels are similar in fasted and control-fed mice despite an immediate drop in serum insulin in fasted mice (Figure 1A bottom row). This might be explained by an early activation of glycogenolysis in the major glucose storing tissues (i.e. muscle and liver [21]). After 12 hours, glycogen stores seem to be depleted and serum glucose levels are significantly lower in fasted mice, suggesting that liver GNG is compensating only partly for the lack of dietary glucose by keeping blood glucose levels around 100 mg/dl (Figure 1A bottom row). Hence, our data on the expression profiles of gluconeogenic genes show an immediate upregulation of hepatic GNG 3 to 6 hours after food withdrawal, despite a delayed reduction of serum glucose levels.

### Increase of liver ketogenesis by three hours after onset of fasting

One substrate for hepatic GNG is glycerol, which is mainly derived from adipose tissue lipolysis where triglycerides are hydrolyzed to glycerol and NEFA. As indicated in Figure 1B, this process results in an increase in serum glycerol and NEFA after 6 hours of fasting. Serum NEFA levels normalize to the control-fed levels at the 24 hour time point, presumably due to increased uptake and utilization of free fatty acids in several tissues (mainly liver and skeletal muscle) while glycogen stores are further depleted. Apart from being a major energy source in times of nutrient deprivation, adipose tissue-derived fatty acids serve as substrate for ketogenesis, the synthesis of ketone bodies (mainly β-hydroxybutyrate and acetoacetate) in the liver. These ketone bodies can be used as secondary energy source by the brain, which cannot utilize fatty acids directly [3]. In our data set, serum β-hydroxybutyrate levels are increasing steadily over 48 hours of fasting compared to ad libitum-fed control mice with the first significant increase at 3 hours (Figure 1B bottom row). This increase is concordant with the upregulation of liver *Hmgcs2* mRNA which is a key enzyme in liver ketogenesis [22] (Figure 1B top row). Interestingly, liver ketogenesis and serum β-hydroxybutyrate are increased hours before blood glucose levels begin to drop (Figure 1A bottom row). This dynamic argues for the involvement of other sensors (for instance gut hormones or insulin and glucagon signaling) detecting the absence of nutritional carbohydrates and signaling to liver to upregulate ketogenesis early in fasting. A recent report introduced an intriguing, novel functional role for ketone bodies. By inhibiting histone deacetylases, β-hydroxybutyrate was shown to induce the expression of genes that protect against oxidative damage in a variety of tissues [23]. In that study, a change in histone deacetylation in kidney was shown at serum β-hydroxybutyrate concentrations greater than 0.5 mM, a level we measured already after 3 hours of food withdrawal. Thus it is conceivable that this β-hydroxybutyrate-mediated mechanism could be activated early on, to set the stage for transcriptional regulation in response to fasting.

### Early fasting-onset upregulation of Ppara target genes

Fgf21 is a recently discovered hormone shown to be a major hub in the hepatic response to fasting by regulating fatty acid and glucose metabolism [24-26]. In our data *Fgf21* mRNA was elevated in liver of fasted animals throughout the 48 hours, peaking at 24 hours (Figure 1B top row). Furthermore, Fgf21 was shown to mediate its effect partly via upregulation of *Ppargc1a*[27], a transcriptional coactivator we found to be highly increased by fasting in accordance with *Fgf21* levels (Figure 1B top row). Ppargc1a in turn increases expression of many fasting response genes by binding and coactivating transcription factors such as Ppara and glucocorticoid receptor [15]. Along these lines, most genes shown in Figure 1 are Ppara target genes (*Pck1, G6pc, Pcx, Gyk, Hmgcs2, Fgf21; see*[18,25,28-30] and references therein) arguing for the central role of this transcription factor during fasting, evident from the phenotype of fasted Ppara knock-out mice [31]. However, the modest changes of liver *Ppara* mRNA levels (Figure 1B top row) are unlikely to cause the strong alterations in Ppara targets (compare for instance to *Fgf21*, a functional Ppara target [25]). Rather the transactivation of Ppara by endogenous ligands (fatty acids and their derivatives [32]), coactivation by Ppargc1a, and synergistic regulation by other fasting-regulated transcription factors (e.g. Foxa2 [33] and Creb [34]) could lead to the magnitude of increase of its target genes.

In summary, comparing expression of key liver fasting genes to serum parameters shows a coherent picture suggesting, in accordance with other recent studies [12,13], a parallel activation of fasting-induced pathways rather than a sequential response as historically believed [1]. This response is activated as early as 3 to 6 hours after food withdrawal and reaches a steady state between 12 and 24 hours. Our data further underlines that Ppara acts as one major fasting hub, by coordinating expression of its target genes. Hence, we provide a detailed view of molecular response kinetics during a 48 hour fasting period in mice allowing one to extrapolate on the timely regulation of the fasting response in liver and in the whole organism.

### Global changes in transcriptome signatures of white adipose tissue, liver, and skeletal muscle in fasted mice

**Figure 2:**
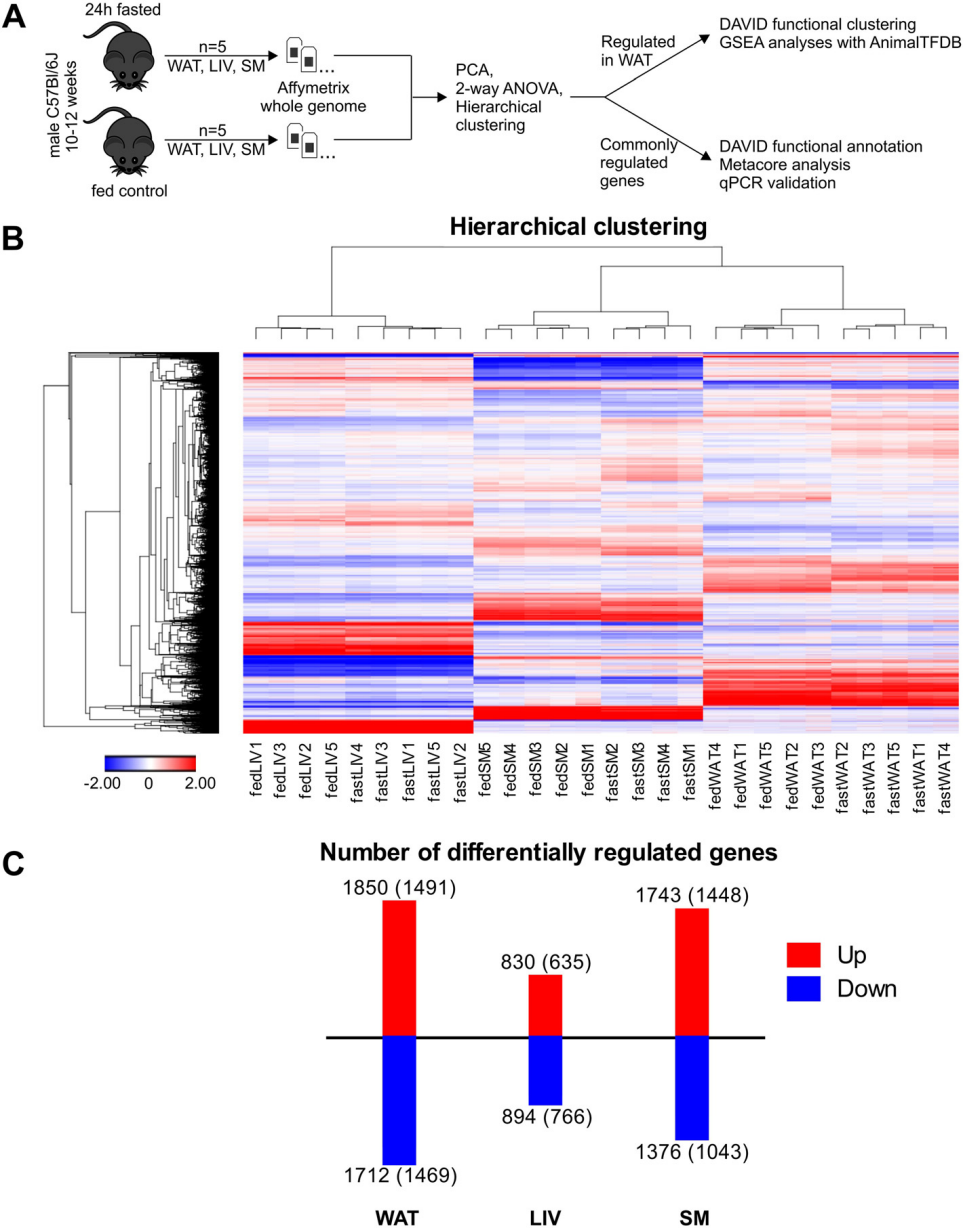
**Overview of microarray experiments in WAT, LIV, and SM 24 hours after onset of fasting. (A)** Experimental design of the transcriptome study (WAT = white adipose tissue, LIV = liver, SM = skeletal muscle, PCA = principal component analysis). **(B)** The heatmap contains about 7000 probe sets differentially expressed ≥ 1.3-fold (FDR5) between fasted and fed in at least one condition. Hierarchical clustering of experiments clusters biological replicates together. Two outlier replicates were identified by principal component analysis and removed from the data set. **(C)** Numbers of up- and downregulated (1.3x, FDR5) microarray probe sets and RefSeq-annotated genes (in parentheses) in WAT, LIV, and SM of mice fasted for 24 hours. Additional file 1 provides detailed expression values for these genes.

Next, we aimed to elucidate RNA abundance responses to fasting in a systematic and genome-wide manner. Most of the parameters determined in Figure 1 show the highest difference between fasted and fed states at 24 hours initiating the experiment, suggesting that the metabolic adaption to fasting has reached a first steady state. For this reason we chose this time point for transcriptome analyses of epididymal white adipose tissue (WAT), liver (LIV), and skeletal muscle (SM) of fasted and control mice (Figure 2A). Tissue-derived mRNA from five fasted and five control mice were hybridized to Affymetrix GeneChip arrays (Mouse Gene 1.1 ST). Two hybridizations (fed liver #4 and fasted skeletal muscle #1) were outliers as determined by principal component analysis and therefore excluded from further analysis. Hierarchical clustering of the remaining microarray data sets showed that experiments strongly cluster by tissue type (Figure 2B). The heatmap contains about 7000 probe sets differentially expressed by ≥ 1.3-fold at a false discovery rate < 5% after multiple testing adjustment (FDR5) in at least one condition. WAT showed the highest number of differentially expressed probe sets between fasting and fed states (1850 upregulated and 1712 downregulated upon fasting, collapsing into 1491 upregulated and 1469 downregulated RefSeq-annotated genes), more than double the number of genes regulated in liver (Figure 2C). Additional file 1 provides the lists of probes expressed more than 1.3-fold with an FDR5 for each of the three tissues.

### Functional annotation clustering of genes regulated in fasted white adipose tissue

**Figure 3:**
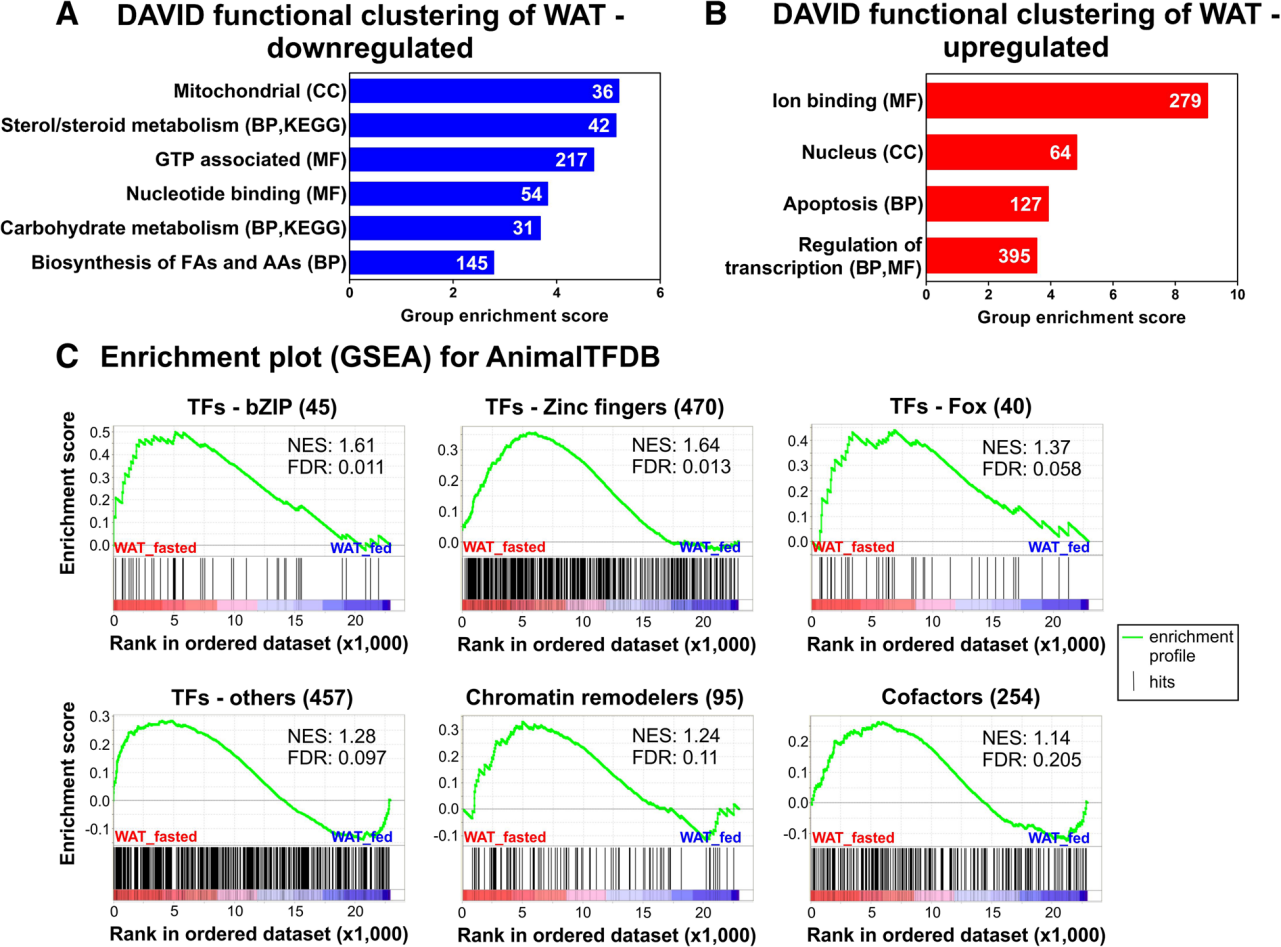
**Functional annotation of genes regulated in WAT 24 hours after onset of fasting reveals activation of transcription regulatory components. (A)** and **(B)** DAVID functional clustering was performed with genes upregulated **(A)** or downregulated **(B)** by fasting (1.3x, FDR5). Shown are the numbers of unique genes (inside of bars) and the enriched clusters (left of bars with contributing DAVID domains in parentheses). The x-axis shows the cluster enrichment scores representing a geometric mean (−log) of p-values of entities in each cluster. Clusters with an enrichment score larger than three are shown. CC = cellular components, MF = molecular functions, BP = biological processes. **(C)** GSEA enrichment plots are generated by ranking all genes in the dataset by expression values, mapping transcription factor (TF) sub-categories (bZIP = basic leucine zippers; Fox = forkhead box), cofactors, and chromatin remodelers, and calculating a cumulative enrichment score (green line). NES = normalized enrichment score, FDR = false discovery rate.

Although the transcriptome signatures of other mouse organs, including liver and muscle, during fasting were extensively investigated [12,13,34] and analyses of adipose tissue transcriptomes during fasting are available (rats [35,36], chicken [37], pigs [38], lactating goats [39]) the current study is, to our knowledge, the first to focus on characterization of the global gene expression response of adipose tissue in fasted mice. Hence, we focused our initial analysis on the WAT dataset and performed functional annotation of the genes up - or downregulated in fasted WAT (1.3x, FDR5) by mapping these lists onto gene ontology (GO; [40]) and KEGG pathways [41] utilizing the DAVID tool [42,43]. To visualize the results we employed the functional clustering option, which combines redundant entities, based on similarity of gene lists, into clusters. Figure 3A and B respectively show the resulting clusters from the lists of “WAT downregulated” or “WAT upregulated” genes together with contributing GO domains in parentheses. The x-axis shows the cluster group enrichment scores representing a geometric mean (−log) of p-values of entities in each cluster. We only show clusters with an enrichment score larger than 3 because they contain at least one entity with a significant p-value after multiple testing correction (<0.05). The detailed results of the functional clustering including terms/pathways contributing to each cluster are given in Additional file 2.

Functional clustering of genes downregulated by fasting in WAT (Figure 3A) yields terms related to “mitochondria” in the cellular component (CC) GO category, indicating suppression of mitochondrial processes in times of energy scarcity. In the GO domain molecular function (MF) we find “GTP-associated” terms and GO terms related to “nucleotide binding”. GO biological processes (BP) and KEGG pathways are found in clusters related to “steroid and sterol metabolism”, “carbohydrate metabolism” and “biosynthesis of fatty acids and amino acids”. Downregulation of steroid and sterol metabolism is analyzed and discussed in more detail later. The cluster of carbohydrate metabolism is mainly comprised of GO terms referring to catabolism of monosaccharides, like hexose or glucose, and might reflect the shift from glucose usage to fatty acid β-oxidation in prolonged fasting. Also the downregulation of fatty acid and amino acid biosynthesis is a plausible reaction of a fasting cell or tissue that needs to suppress energy consuming processes which increase anaplerotic pressure.

### Genes upregulated in fasted white adipose tissue are strongly connected to transcriptional regulation

Genes that are upregulated in fasted WAT (Figure 3B) show the highest enrichment score for the GO MF category related to “ion binding”, which is a rather general and unspecific term. However, we find a cluster containing 127 genes that relate to “apoptosis” and “cell death” indicating that apoptotic pathways are activated during fasting in WAT. Finally, we find high enrichment in a cluster containing “nucleus”-related GO CC terms as well as a cluster with terms referring to “regulation of transcription” (BP and MF), both of which indicate a strong positive influence of fasting on the transcriptional program of adipose tissue. This is consistent with WAT being the tissue with the highest number of differentially regulated genes when compared to LIV and SM (Figure 2C). To substantiate this finding we tested whether the genes in the upregulated list are enriched for transcriptional regulator molecules such as transcription factors, cofactors and chromatin remodelers as defined by the manually curated AnimalTFDB [44]. As a control we also tested for enrichment in the list of genes downregulated by fasting in WAT which did not map to “regulation of transcription”-associated GO terms in our DAVID analysis. As shown in Additional file 3 the WAT upregulated list contains at least double the number of transcriptional regulators, compared to the WAT downregulated list (e.g. 122 vs. 50 transcription factors), and comprises 9%, 12%, and 16% of annotated mouse transcription factors, cofactors, and chromatin remodelers, respectively. Additional file 3 also lists the expression values and the annotations of regulated genes in WAT according to the transcription factor subclasses defined in AnimalTFDB. Based on ranking of the entire data set, the gene-set enrichment analysis tool [45] performs a similar analysis and yields a significant enrichment for the sub-categories (all but HOX factors) of transcription factors from AnimalTFDB for upregulated genes (see Figure 3C and Additional file 3). These transcription factor families contained 43 genes encoding zinc finger transcription factors (including kruppel-like factors Klf4, Klf9, and Klf15), 9 genes encoding basic leucin zippers (bZIP), and 6 forkhead box genes that are upregulated in fasted WAT (see Additional file 3). Among the transcription factor-encoding genes with the highest upregulation by fasting (14-fold) we find *Irf4*, which has been shown to tip the scales between lipogenesis and lipolysis in the latter direction in a fasting-induced manner [46]. Consequently, fat-specific Irf4 knock-out mice are deficient in lipolysis and show increased adiposity [46]. Furthermore, genes encoding the transcription factors *Zim1* and *Peg3* are massively upregulated by fasting (21-fold and 6-fold, respectively). Interestingly, these two genes are adjacently located at an imprinted region on mouse chromosome 7. The Peg3 knock-out mouse model develops increased adiposity despite lower food intake. This was attributed to developmental deficiencies that lead to aberrant leptin signaling in the hypothalamus [47]. However, our data suggest a direct involvement of the Peg3/Zim1 locus in adipose tissue biology.

The present study is, to our knowledge, the first to focus on the characterization of the transcriptome response to fasting of WAT in mice. Surprisingly, we do not find GO mapping to the biological process “lipid catabolism” which would be expected to be prominent during fasting in WAT. The absence thereof could be explained by abundant posttranscriptional regulation of lipolysis by kinases such as protein kinase A [48] and AMP-activated protein kinase [6] which are not reflected at the transcript level. Instead our analyses specifically reveal an unexpected upregulation of cell death pathways as well as a strong enrichment of transcriptional regulators among genes activated by fasting in WAT.

### p53 signaling as top ranking pathway in the fasting response of major metabolic tissues

**Figure 4:**
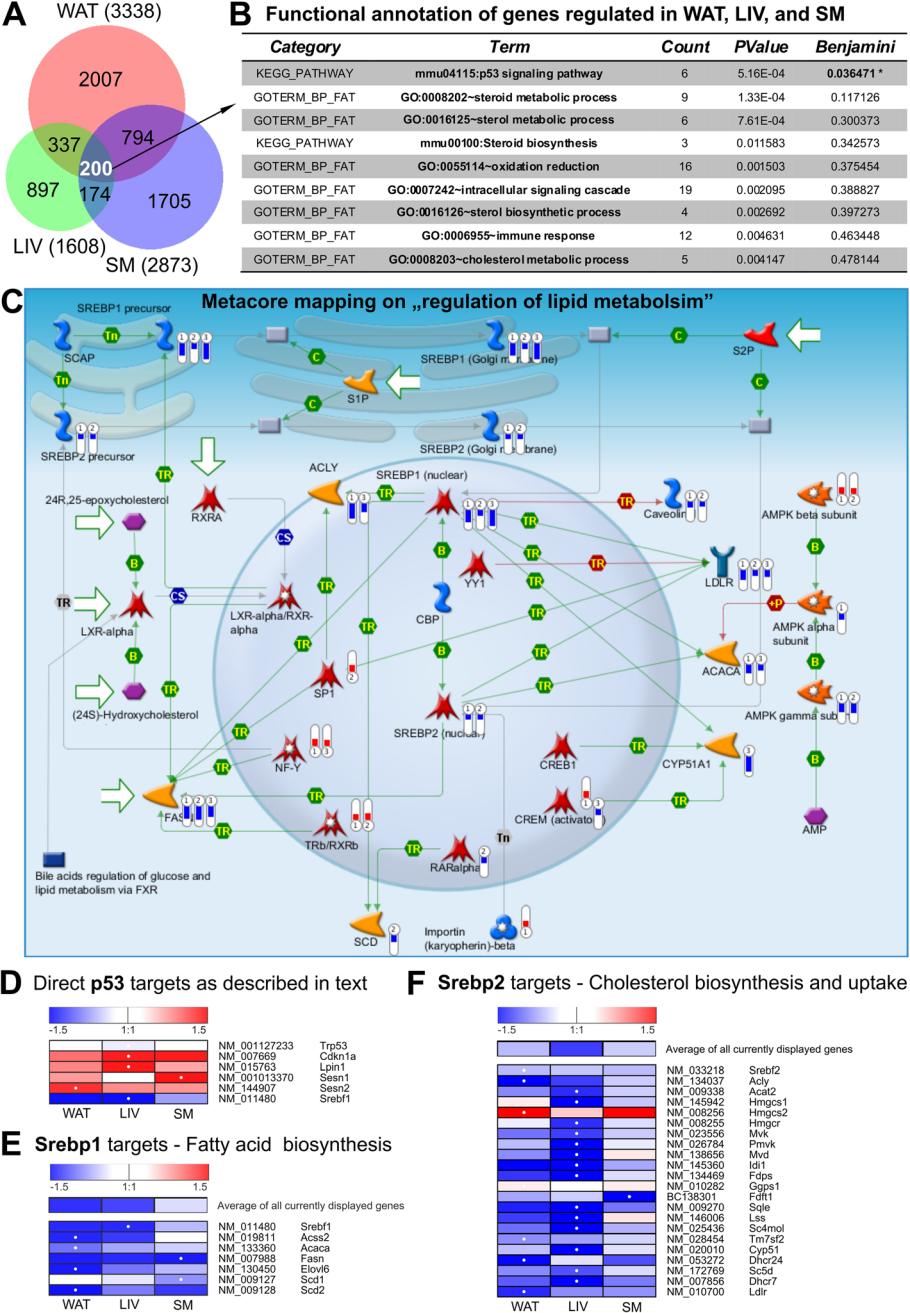
**Analyses of differentially expressed genes regulated by fasting in all three tissues revealing an involvement of p53 and Srebp signaling pathway. (A)** Venn diagram of genes differentially regulated (1.3x, FDR5) in WAT, LIV, and SM and their intersections. **(B)** Two hundred genes differentially regulated in all three tissues were submitted to DAVID functional annotation using GO terms and KEGG pathways. Count shows the number of mapped genes and Benjamini-Hochberg’s correction was used for adjusting p-value for multiple testing. **(C)** Metacore mapping as obtained from analysis with the commonly regulated gene list. The thermometers display expression levels of mapped genes (1: WAT, 2: SM, 3: LIV). **(D)** Heatmap of p53 target genes discussed in the text. Values are log2 transformed. White dots mark highest expression between the 3 tissues. **(E)** and **(F)** Manually compiled heatmaps indicating expression levels of genes comprising the FA **(E)** and cholesterol biosynthesis **(F)** pathways. Values are log2 transformed. White dots mark highest expression between the 3 tissues.

By producing and exporting glucose, fatty acids, glycerol, and ketone bodies WAT, LIV, and SM represent the organs mainly responsible for energy homeostasis during a fasting period in mammals [1,21]. To reveal common pathways we focused on 200 genes that were regulated by fasting in all three tissues (Figure 4A, termed “common list” in the following). Table 2 lists these genes ranked by decreasing average expression level. Mapping the common list to GO biological processes and KEGG using DAVID yielded one KEGG pathway as significantly enriched after multiple testing correction: the p53 signaling pathway (Figure 4B). Additionally, an independent analysis with Metacore (GeneGo, Inc.) focusing on networks overrepresented in the common list reveals the p53 node as the second highest scoring network hub (see Additional file 4 for genes that constitute this network; the highest scoring hub was the ubiquitous transcription factor Sp1). The p53 transcription factor is known to regulate a number of tumor suppressor pathways including cellular senescence, apoptosis, and DNA repair in response to various stressors [49,50]. About 50% of human cancers carry mutations in the p53 gene [51]. However, evidence is now accumulating that p53 plays a prominent role in (lipid) metabolism as well [52,53]. There is no direct evidence of a physiological importance of p53 signaling in fasting, but some studies report an activation of p53, and an induction of its target genes, upon glucose deprivation in cultured cells [54,55]. In our dataset we observe a host of p53 target genes apparently regulated by fasting, and a heatmap of the genes described below is shown in Figure 4D. For instance, the canonical p53 target *Cdkn1a*[49], a cell cycle regulator better known as p21, is one of the top ranked genes in Table 2 and is the gene with the highest fasting-induced upregulation in liver (>10x). Another example is lipin-1 encoded by the gene *Lpin1*, which has recently been described as a novel p53 target [55]. In our data *Lpin1* is upregulated in WAT, LIV, and SM by fasting. Depending on its subcellular location lipin-1 can act to enhance fatty acid oxidation by interacting with Ppara and Ppargc1a (in the nucleus) [56] or, as a phosphatidate phosphatase in the cytosol, to perform a key step in triglyceride biosynthesis [57]. Being upregulated by fasting suggests activity of its nuclear form to drive oxidation of fatty acids in lipid storing tissues. Other p53 targets are the Sestrins, *Sesn1* (upregulated by fasting in WAT and SM) and *Sesn2* (upregulated in all 3 tissues), which have recently been shown to be induced by fasting in liver and to protect it from oxidative damage in a fasting/refeeding scenario [58]. Interestingly, Sen et al. report that p53 physically interacts with Ppargc1a [59], which we show to be strongly upregulated in liver (Figure 1B) by qPCR as well as in our microarray study in WAT and LIV (it is downregulated, however, in SM). In their study Sen et al. show that Ppargc1a can bind and thereby direct p53 to the promoters/enhancers of pro-arrest as well as metabolic target genes. Thus, this interaction of p53 and its coactivator Ppargc1a could lead to a tissue-specific coordination of p53 to target genes relevant for a proper response.

**Table 2:** Fold difference in expression (fasted/fed) of transcripts regulated in WAT, LIV, and SM by fasting

**UNIQID**	**NAME**	**WAT**	**LIV**	**SM**	**UNIQID**	**NAME**	**WAT**	**LIV**	**SM**
NM_029083	Ddit4	7.86	4.46	17.72	NM_133904	Acacb	2.74	−2.05	1.40
NM_007669	Cdkn1a	2.02	10.20	4.43	NM_024479	Wbscr27	1.48	−1.43	2.02
NM_007679	Cebpd	3.36	2.29	8.54	NM_016972	Slc7a8	2.03	−1.72	1.74
NM_010220	Fkbp5	3.40	5.84	4.19	NM_019873	Fkbpl	−1.33	1.33	2.03
BC096647	E230001N04Rik	2.50	4.54	5.11	NM_008580	Map3k5	1.51	1.72	−1.40
NM_183187	Fam107a	3.96	3.56	3.27	NM_172648	Ifi205	1.66	−1.95	2.03
NM_008817	Peg3	6.05	2.53	2.10	NM_133748	Insig2	1.61	1.48	−1.36
NM_010559	Il6ra	2.91	2.16	4.47	NM_009104	Rrm2	−1.39	1.65	1.47
NM_016693	Map3k6	3.06	2.05	3.96	NM_009653	Alas2	1.65	−1.49	1.53
NM_027560	Arrdc2	2.74	1.65	4.48	NM_027442	Ddo	1.65	1.31	−1.33
NM_018881	Fmo2	2.51	3.18	2.75	NM_133955	Rhou	1.38	−1.50	1.70
NM_001159367	Per1	2.51	3.17	2.66	NM_010197	Fgf1	−1.33	1.47	1.37
NM_011704	Vnn1	4.48	1.78	1.96	NM_001081401	Adamts3	1.48	1.38	−1.38
NM_139306	Acer2	4.07	1.38	2.58	NR_004446	Gm7035	1.49	−1.52	1.49
NM_001077348	Plin5	2.06	2.76	3.15	NM_028595	Ms4a6c	1.41	−1.53	1.40
NM_029415	Slc10a6	2.74	1.89	3.31	NM_029620	Pcolce2	1.32	−1.40	1.33
NM_133765	Fbxo31	3.71	2.46	1.60	NM_001081317	Anubl1	1.44	−1.68	1.32
NM_201256	Eif4ebp3	3.08	3.18	1.34	NM_029494	Rab30	−1.37	3.28	−1.63
NM_001033324	Zbtb16	1.54	3.91	2.02	NM_027868	Slc41a3	−1.58	3.50	−2.49
NM_030166	Galntl2	3.09	1.63	2.67	NM_008063	Slc37a4	−1.38	1.93	−1.36
ENSMUST00000059018	1110003O08Rik	3.57	2.31	1.46	NM_029967	Adamtsl1	1.53	−1.34	−1.34
NM_013602	Mt1	1.79	3.05	2.41	NM_177471	Ccdc69	1.61	−1.34	−1.45
ENSMUST00000039517	Syde2	4.10	1.60	1.51	NM_026250	Zh2c2	−1.30	1.41	−1.37
NM_009994	Cyp1b1	3.19	1.46	2.35	NM_178111	Trp53inp2	−1.63	1.36	−1.39
NM_001013780	Slc25a34	1.83	2.19	2.98	NM_009255	Serpine2	−1.67	1.58	−1.60
NM_011817	Gadd45g	2.53	2.33	2.07	NM_001005341	Ypel2	1.39	−1.50	−1.61
NM_010706	Lgals4	2.49	2.68	1.69	NM_177351	C630028N24Rik	−1.96	1.39	−1.37
NM_011366	Sorbs3	1.92	2.14	2.62	NM_026524	Mid1ip1	−1.97	−2.03	1.81
NM_011977	Slc27a1	2.27	2.99	1.35	NM_011943	Map2k6	−2.14	1.68	−2.20
NM_015763	Lpin1	1.83	2.87	1.59	NM_027950	Osgin1	−1.84	−3.45	2.46
NM_025404	Arl4d	1.58	1.84	2.84	ENSMUST00000100735	Gm10387	−1.68	1.59	−2.85
NM_153574	Fam13a	2.93	1.71	1.50	ENSMUST00000111704	Rassf8	−1.31	−1.31	−1.47
NM_001113283	BC031353	2.44	1.95	1.67	NM_011063	Pea15a	−1.32	−1.45	−1.36
ENSMUST00000095691	Gm9766	1.56	1.57	2.88	BC016078	4931406C07Rik	−1.33	−1.35	−1.47
NM_001077364	Tsc22d3	2.23	2.24	1.53	NM_172928	Dclk3	−1.53	−1.32	−1.30
NM_144907	Sesn2	2.50	1.72	1.64	NM_025901	Polr3k	−1.40	−1.40	−1.36
BC019494	Fam134b	2.28	1.48	1.97	NM_016701	Nes	−1.39	−1.39	−1.45
NM_001004468	Tacc2	1.75	1.48	2.32	NM_028651	Tmtc4	−1.47	−1.41	−1.36
NM_001081005	1500012F01Rik	1.85	1.51	2.19	NM_009448	Tuba1c	−1.35	−1.66	−1.32
NM_001037709	Rusc2	1.59	1.72	2.02	NM_026102	Daam1	−1.46	−1.31	−1.57
NM_026646	Slc25a22	1.34	1.52	2.26	NM_025972	Naaa	−1.52	−1.31	−1.54
NM_001033238	Cblb	1.60	1.43	2.08	NM_008150	Gpc4	−1.71	−1.32	−1.35
NM_001033352	Klhl21	1.94	1.41	1.74	NM_019440	Irgm2	−1.58	−1.39	−1.42
NM_133670	Sult1a1	1.71	1.45	1.91	NM_183180	Tspan18	−1.53	−1.36	−1.49
NM_009760	Bnip3	1.57	1.80	1.69	ENSMUST00000108947	Gm14403	−1.34	−1.60	−1.46
NR_002840	Gas5	1.53	1.36	2.17	NM_001037170	Tomm40l	−1.69	−1.46	−1.31
NM_023184	Klf15	1.62	1.56	1.80	NM_011654	Tuba1b	−1.34	−1.72	−1.39
NM_029166	Uhrf1bp1l	1.94	1.54	1.48	NM_001111110	Cmah	−1.46	−1.49	−1.55
NM_153075	Catsper2	1.46	2.18	1.31	NM_178607	Rnf24	−1.70	−1.39	−1.45
NM_007876	Dpep1	1.71	1.78	1.44	NM_009829	Ccnd2	−1.40	−1.53	−1.66
BC150711	AI607873	1.56	1.38	1.98	NM_007486	Arhgdib	−1.52	−1.69	−1.40
NM_023635	Rab27a	1.33	1.60	2.00	NM_026740	Slc46a1	−1.99	−1.32	−1.33
NM_030697	Kank3	1.70	1.61	1.60	NM_011505	Stxbp4	−1.38	−1.44	−1.82
NM_001001883	Hecw2	1.86	1.62	1.38	NM_028372	Mblac2	−1.63	−1.58	−1.44
NM_019765	Clip1	1.45	1.39	2.02	NM_001080707	Gpr155	−1.82	−1.34	−1.52
ENSMUST00000077293	Gm5785	1.38	1.67	1.79	NM_025855	Echdc1	−1.47	−1.96	−1.32
NM_146085	Apbb3	1.52	1.54	1.79	NM_026170	Ergic1	−1.93	−1.34	−1.49
NM_133865	Dclre1b	1.76	1.41	1.64	NM_009388	Tkt	−1.65	−1.57	−1.55
NM_001081417	Chd7	1.31	2.01	1.46	NM_007984	Fscn1	−1.71	−1.58	−1.49
NM_022331	Herpud1	1.67	1.56	1.55	NM_133871	Ifi44	−1.66	−1.70	−1.44
NR_027965	2310061J03Rik	1.79	1.49	1.48	NM_152804	Plk2	−1.78	−1.41	−1.66
NM_020253	Nrxn2	1.87	1.53	1.33	NM_024257	Hdhd3	−1.73	−1.56	−1.59
AK051045	Snhg1	1.51	1.39	1.79	NM_008538	Marcks	−1.80	−1.52	−1.57
NM_001033528	Usp36	1.37	1.42	1.89	NM_178389	Gale	−1.73	−1.89	−1.31
NM_175480	Zfp612	1.65	1.56	1.41	NM_008788	Pcolce	−1.64	−1.65	−1.67
NM_011728	Xpa	1.50	1.45	1.62	NM_178079	Pm20d1	−1.97	−1.44	−1.56
NM_197986	Tmem140	2.36	−1.33	3.52	NM_145545	Gbp6	−1.87	−1.68	−1.48
NM_007705	Cirbp	1.42	1.79	1.34	NM_178869	Ttll1	−1.92	−1.40	−1.75
NM_194342	Unc84b	1.55	1.39	1.58	NM_013867	Bcar3	−2.20	−1.41	−1.47
NM_144788	Hectd1	1.35	1.36	1.80	NM_007934	Enpep	−2.15	−1.48	−1.47
NM_012050	Omd	1.38	1.72	1.39	NM_010357	Gsta4	−1.74	−1.71	−1.66
NM_026159	Retsat	1.35	1.59	1.53	XR_030785	Gm7172	−1.73	−1.68	−1.77
NM_027468	Cpm	1.77	1.37	1.31	NM_008209	Mr1	−2.38	−1.51	−1.33
NM_019713	Rassf1	1.52	1.33	1.55	NM_021273	Ckb	−2.12	−1.64	−1.50
NM_013863	Bag3	1.42	1.55	1.42	NM_009825	Serpinh1	−2.03	−1.51	−1.76
NM_199299	Phf15	1.34	1.43	1.56	NM_010260	Gbp2	−1.87	−1.68	−1.76
NM_026929	Chac1	3.69	−1.68	2.22	XR_030619	LOC100044416	−1.90	−1.72	−1.70
NM_026448	Klhl7	1.49	1.31	1.43	NM_018734	Gbp3	−2.11	−1.37	−1.87
ENSMUST00000052189	B230317F23Rik	1.40	1.38	1.43	NM_009425	Tnfsf10	−1.79	−1.40	−2.19
NM_026493	Cspp1	1.35	1.35	1.49	NM_008695	Nid2	−2.25	−1.61	−1.55
NM_001081101	4933407H18Rik	1.47	1.36	1.33	NM_009930	Col3a1	−1.70	−1.79	−1.93
NM_001033208	Gcom1	1.50	1.34	1.32	NM_020282	Nqo2	−2.08	−1.75	−1.61
NM_198020	Trmt1	1.46	1.33	1.31	NM_010700	Ldlr	−2.41	−1.60	−1.55
NM_026160	Map1lc3b	1.41	1.34	1.34	NM_007631	Ccnd1	−1.40	−2.12	−2.31
NM_138953	Ell2	1.35	1.41	1.31	ENSMUST00000071723	Akap2	−2.75	−1.44	−1.78
NM_009349	Inmt	3.33	−1.74	2.47	NM_054098	Steap4	−2.77	−1.67	−1.59
NM_183288	Sh3d20	1.31	1.37	1.35	NM_025558	Cyb5b	−2.68	−1.84	−1.59
NM_019861	Ctsf	1.35	1.33	1.32	NM_025436	Sc4mol	−1.81	−3.09	−1.38
NM_178873	Adck2	1.32	1.32	1.31	NM_007856	Dhcr7	−1.96	−2.81	−1.72
NM_026779	Mocos	1.37	−1.37	3.29	NM_011498	Bhlhe40	−2.09	−1.41	−3.05
NM_010442	Hmox1	1.48	−1.47	2.74	NM_011923	Angptl2	−1.80	−1.47	−3.30
NM_007521	Bach2	1.90	−1.34	2.16	NM_027249	Tlcd2	−3.24	−2.08	−1.42
NM_009346	Tead1	2.60	1.35	−1.47	NM_011579	Tgtp	−2.17	−1.95	−2.95
BC049153	2610024B07Rik	2.63	1.30	−1.55	NM_010255	Gamt	−5.02	−1.73	−1.53
NM_026433	Tmem100	−1.98	2.32	2.03	NM_145360	Idi1	−2.57	−4.38	−1.43
NM_018760	Slc4a4	2.22	1.49	−1.36	NM_053078	D0H4S114	−3.72	−2.12	−2.57
AY140896	LOC433762	−1.42	2.09	1.60	NM_011480	Srebf1	−3.01	−3.85	−1.56
NM_023617	Aox3	2.38	−1.53	1.40	NM_007988	Fasn	−3.01	−2.69	−3.32
NM_010831	Sik1	1.83	−1.56	1.92	NM_009270	Sqle	−2.49	−5.00	−1.77
NM_025638	Gdpd1	2.11	1.37	−1.40	NM_009128	Scd2	−9.70	−1.81	−2.40

Genes with >1.3x change (FDR5) in all investigated tissues.

### Fasting-mediated downregulation of Srebp pathways is common to major metabolic tissues

It was reported that p53 is capable of suppressing the promoter of the gene encoding the transcription factor sterol-regulatory element binding protein 1 (Srebp1 [60]). The Srebp family consists of three members: Srebp1a and Srebp1c, both transcribed from the same gene *Srebf1*, with Srebp-1c being the predominant isoform to regulate lipogenesis in metabolic tissues, such as WAT and LIV; and Srebp2, transcribed from the *Srebf2* locus and responsible for regulation of sterol metabolism [61-63]. Consistent with these reports, a Metacore analysis on the common list delivers “Regulation of lipid metabolism” as top ranking pathways with the Srebp family of transcription factors in its center (Additional file 5). As mapped in Figure 4C, we find *Srebf1* and *Srebf2* downregulated in all three tissues (LIV Srebp2 is not mapped because it shows a significant 1.26x downregulation, not meeting our 1.3x cut-off). Concordantly, established Srebp1 downstream genes, coding for enzymes which encompass the fatty acid biosynthesis pathway like *Acss2*, *Acaca*, *Fasn*, *Scd1*, and *Scd2*[64], are robustly down-regulated by fasting in our data (Figure 4E, Table 2). Further, DAVID analyses for the set of commonly regulated genes (Figure 4B) as well as for regulated liver genes (Additional file 6) yields GO biological processes that refer to steroid metabolism and, more specifically, to cholesterol biosynthesis. As a pivotal regulator of cholesterol homeostasis in cells, Srepb2 mediates its effects by control of *de novo* synthesis and/or by regulation of cholesterol import [65]. To regulate *de novo* synthesis, Srebp2 (and to some extent also Srebp1) binds to promoters of most of the enzymes in the cholesterol biosynthesis pathway [63]. We find that transcripts for most of these enzymes are significantly downregulated or show at least a trend to downregulation (Figure 4F). The internalization of cholesterol is regulated by Srebp-mediated transcription of the LDL-receptor Ldlr (down in all three tissues). Figure 4F shows a heatmap of these Srebp2 target genes [63] and impressively demonstrates reduction of transcripts for nearly all components of the cholesterol biosynthesis and uptake pathway in the investigated tissues. Given the extensive and intricate networks that, depending on sterol or nutrient availability, post-translationally regulate processing and activation of membrane-bound SREBPs [66], our finding that fasting broadly regulates Srebp-dependent pathways already at the transcriptional level is rather surprising. Especially in liver, this downregulation of cholesterol biosynthesis in combination with the upregulation of *Hgmcs2* (Figure 1A and Figure 4F), which condenses acetoacetyl CoA and acetyl CoA to β-hydroxy β-methylglutaryl CoA, hints to a diversion of substrate from sterol synthesis to ketone body synthesis.

Hence, based on our analyses on genes regulated in WAT, LIV, and SM, we hypothesize a shared mechanism that responds to the fasting stimulus in all three tissues: The p53 signaling pathway is activated by fasting in WAT, LIV, and SM (perhaps via AMPK as described in [67]). Coactivators like Ppargc1a direct p53 to promoters/enhancers of genes targeted for transcription activation or repression when nutrients are lacking. Upregulation of p53 targets such as Lpin1 might contribute to the shift of fasted tissues to fatty acid oxidation to provide energy substrates. The observation that p53 knock-out mice are incapable of inducing liver fatty acid oxidation upon fasting [54] underlines this hypothesis. In addition, a p53-mediated downregulation of Srebp1 is followed by a repression of fatty acid biosynthesis (Figure 4C and E). However, we note that other (parallel) pathways that are regulated at the post-translational level, and therefore not reflected in RNA levels, could be responsible for some of the observed effects, such as downregulation of Srebp transcripts. One conceivable example is the activation of AMPK by fasting which, via subsequent deactivation of mechanistic target of rapamycin complex 1 (mTORC1), could be responsible for the decrease in Srebp mRNA [62]. However, based on our analyses we propose a novel and potentially crucial role for p53 in fasting, which eventually could manifest in profound transcriptional changes in several metabolic pathways. Although functional proof of this mechanism is necessary, several reports support our model [52,54,55,58-60].

### Validation of expression of top-ranked genes commonly regulated by fasting in WAT, LIV, and SM

**Figure 5:**
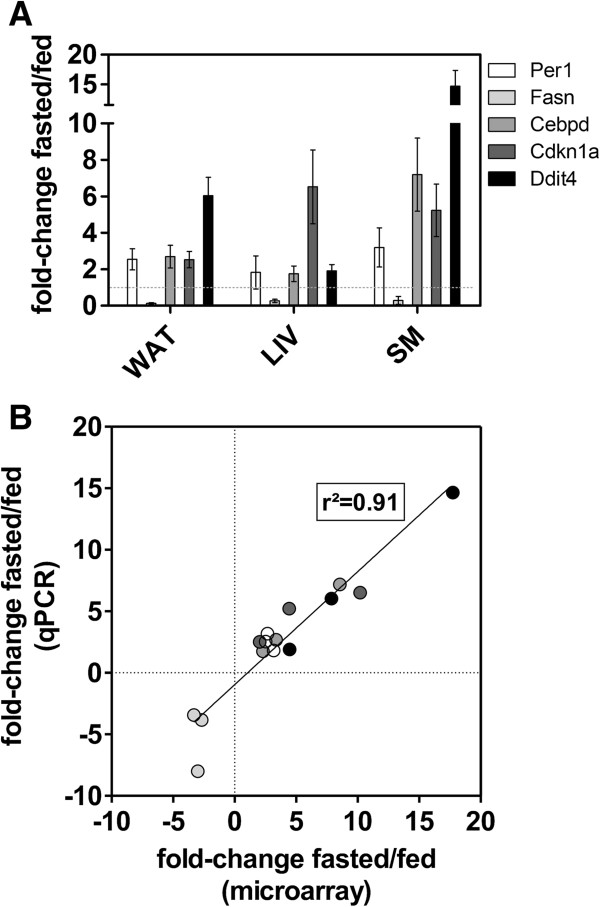
**Validation of selected candidates by qPCR. (A)** Five genes differentially regulated in WAT, LIV, and SM were selected for qPCR validation of microarray results (n = 5). All differences indicated between fasted and control fed are significant with a p-value < 0.05 (two-tailed, unpaired student’s t-test). **(B)** Scatterplot of expression levels measured with qPCR and microarrays. Goodness of fit (r^2^) is calculated with linear regression. Grey shading is according to legend in A.

To validate the microarray data by means of qPCR we selected the top 3 genes from Table 2 (highest average expression across WAT, LIV, and SM: *Ddit4*, *Cdkn1a*, and *Cebpd),* none of which have previously been functionally described in the context of fasting in mice, as well as *Per1* and *Fasn*, known responders to food deprivation and thereby positive controls (Figure 5). Per1 is an essential regulator in the core clock machinery of circadian rhythm [10,68] and, in LIV and AT at the beginning of the light phase, it was previously shown that its expression is higher in fasting compared to control fed mice [69,70]. This difference is due to a circadian phase shift that takes place during an extended fasting period [69]. As mentioned above Fasn has long been known to be a downstream target gene of Srebp1 and is downregulated due to the drop in Srebp1 levels during fasting [71,72]. The basic-leucin zipper transcription factor Cebpd has been described in a number of cellular contexts, such as osteogenesis and adipogenesis (reviewed in [73]). Cebpd expression is known to respond to glucocorticoids and to increased cAMP levels, both of which could explain its upregulation upon fasting. Cdkn1a (p21) as a major p53 target gene, is mainly described as a cell cycle and apoptosis regulator that inhibits cyclin-dependent kinases [74] and has no known role in fasting. Finally, Ddit4, a gene initially reported to be readily induced by dexamethasone [75] as well as upon certain cellular stresses (e.g. hypoxia [76] and exercise in muscle [77]), shows the highest extent of upregulation in WAT and SM in the common list (Table 2). Interestingly, it has also been described as a p53 target gene [78], which led us to further investigate it (see below). Hence, we confirmed fasting-mediated regulation of all genes selected for qPCR validation in all three tissues (Figure 5A) and show a strong correlation with the microarray measurements for all these genes (Figure 5B, r^2^ = 0.91). This introduces three intriguing and novel players in the response to fasting.

### Ddit4 is fasting-induced in WAT, LIV, and SM and is inducible by p53 activation in cultured adipocytes

**Figure 6:**
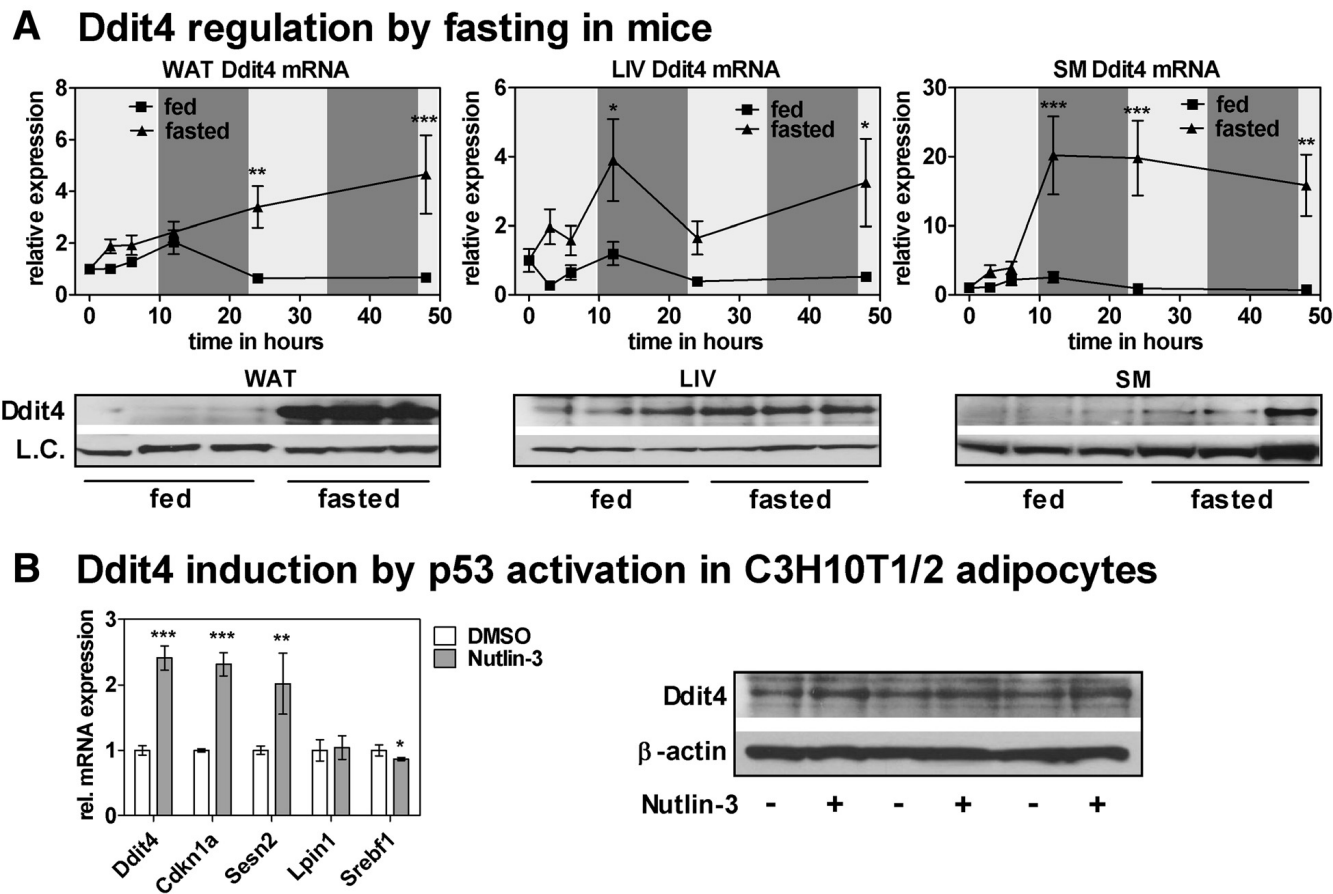
**Ddit4 is induced during fasting and upregulated by p53 activation in cultured adipocytes. (A)***In vivo* regulation of Ddit4 in fasted and fed mice. Relative expression values from qPCR measurements (n = 5) were normalized to 36b4 expression and the value for the first time point was set to 1. Significance was determined with a 2-way ANOVA followed by a Bonferroni posttest to determine significance for the single time points (*p < 0.05; **p < 0.01; ***p < 0.001). Western blots were performed with tissue samples from six month old mice fed a chow diet (fed) or fasted overnight (fasted). Samples are from three littermates each. The following proteins served as loading controls (L.C.): β-actin for WAT and LIV, β-tubulin for SM. **(B)** 10 μM Nutlin-3, a specific p53 activator, was used to treat mature C3H10T1/2 adipocytes (day 7 of differentiation) for 6 hours. qPCR mRNA measurements (n = 3) were normalized to 36b4 expression and related to control cells (treated with DMSO). A two-tailed, unpaired student’s t-test was used to determine statistical significance (*p < 0.05; **p < 0.01; ***p < 0.001). The western blot shows data from three independent replicates treated with Nutlin-3 (+) or DMSO (−) using β–actin as loading control.

To investigate p53 signaling as a common fasting regulator in WAT, LIV, and SM, we focused on DNA damage-induced transcript 4 (Ddit4, aliases: Redd1, Dig2, Rtp801), the top ranking gene in Table 2 which has been described as functional p53 target gene [78]. In all three tissues investigated, Ddit4 mRNA is upregulated at latest by 24 hours after onset of fasting and overnight fasting is sufficient to increase Ddit4 protein levels (Figure 6A), which has been shown by others in gastrocnemius muscle of rats [79]. In our data, differences in the magnitude of fasting-induction between the mRNA and protein level (compare SM mRNA at the 24 h time point with protein levels in Figure 6A) could be explained by the fact that Ddit4 protein stability is highly regulated in different cell systems [80-82]. However, especially in adipose tissue Ddit4 protein seems to be stably induced when mice are fasted. To show that Ddit4 can be directly induced by p53 in adipocytes, we treated mature C3H10T1/2 adipocytes for 6 hours with Nutlin-3, a specific p53 activator [83]. Nutlin-3 treatment led to an increase of Ddit4 mRNA similar to the canonical target Cdkn1a (Figure 6B). Most importantly, Ddit4 protein was also stably induced in all replicates (Figure 6B). In addition, the p53 targets Sesn2 and Srebf1 were regulated by Nutlin-3 in a way similar to the in vivo fasting situation (compare their expression in Figure 6B and 4D). Hence, Ddit4 is stably induced by fasting and upregulated by p53 activation in cultured adipocytes.

### Overexpression of Ddit4 is sufficient to increase lipolysis in cultured adipocytes

**Figure 7:**
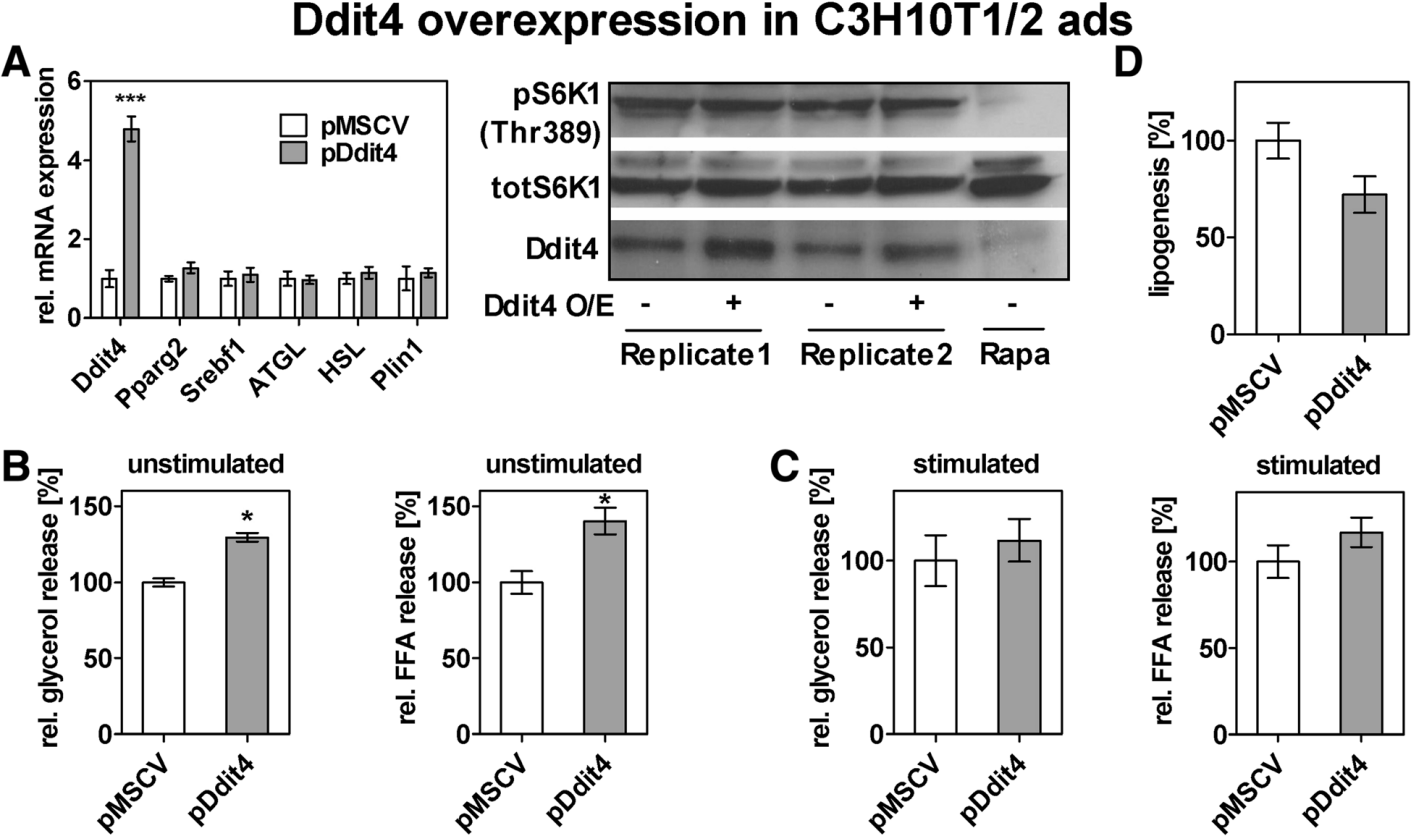
**Transient overexpression of Ddit4 is sufficient to induce lipolysis in cultured adipocytes. (A)-(D)** C3H10T1/2 cells were differentiated to adipocytes for 7 days. Cells were detached and electroporated in the presence of either empty vector (pMSCV) or Ddit4 overexpression vector (pDdit4). **(A)** 48 hours later cells were harvested to measure mRNA (left qPCR) and protein (right western blots), and supernatants were collected for glycerol and FFA assay. qPCR measurements (n = 3) were normalized to 36b4 expression and related to control cells and a two-tailed, unpaired student’s t-test was used to determine statistical significance (***p < 0.001). Western blot shows 2 biological replicates and a control treated with 100 nM rapamycin (Rapa). **(B)** and **(C)** Glycerol and FFA in cell supernatants were determined, normalized to protein content and related to measurements from control cells to yield the relative release caused by Ddit4 overexpression in unstimulated **(B)** and isoproterenol-stimulated (1 μM, 1 hour) **(C)** conditions. Significance was determined by a one-sample t-test (* p < 0.05). **(D)** Electroporated C3H10T1/2 adipocytes were incubated overnight with medium containing ^14^C-labeled deoxy-glucose. Incorporated radioactivity was counted in the lipid extract and related to controls (n = 3). A statistically significant difference was not detected by a one-sample t-test.

In a recent report Ddit4 was shown to be involved in lipid metabolism in adipocytes signaling via the mTORC1 pathway [81]. Also in other studies, Ddit4 has been repeatedly described as a negative regulator of mTORC1 in a variety of cell types [84,85]. Interestingly, in the context of starvation, the nutrient-sensitive mTORC1 pathway needs to be suppressed for the proper fasting response in liver [86] and its suppression induces lipolysis in adipocytes [87]. Hence, we examined whether upregulation of Ddit4 promotes lipolysis in adipocytes by inhibiting mTORC1 activity. For this we transiently overexpressed Ddit4 in differentiated C3H10T1/2 adipocytes (Figure 7A) and determined glycerol and FFA in the medium as a measure of lipolysis. Indeed, we observed a ~30% increased glycerol release and a ~40% increased FFA release from Ddit4 overexpressing cells compared to the empty vector control (Figure 7B), while expression of genes in the lipolytic pathway remained unchanged (Figure 7A). Upon β-adrenergic stimulation (1 hour 1 μM isoproterenol) the increase in lipolysis upon Ddit4 overexpression was still evident, although not statistically significant (Figure 7C). However, as assayed by phosphorylation of the downstream target S6K1 at threonine 389, mTORC1 activity was unchanged despite effective overexpression of Ddit4 (western blot in Figure 7A). An antibody against total S6K1 protein served as loading control and cells treated with rapamycin, a potent exogenous mTORC1 inhibitor, as control for phosphorylation-specific S6K1 antibody. Others have reported that, in SM, dexamethasone-mediated Ddit4 increase leads to reduced mTORC1 signaling [88], but, judging from our data, in adipocytes Ddit4-mediated lipolysis seems to be independent of mTORC1 activity. This is consistent with the observation that the Ddit4-mTORC1 axis is functional in some cell types but not in others [75]. Further, as knock-down of Ddit4 was reported to decrease insulin-stimulated de-novo lipogenesis in adipocytes [81], we wanted to rule out that the observed increase in lipolysis upon Ddit4 overexpression is merely an effect of increased lipogenesis and with that higher lipid content per se. In addition to an unchanged phenotype as shown by mRNA expression of adipocyte-specific genes (Figure 7A), we did not detect an increase in the incorporation of radio-labeled glucose into total lipids in Ddit4 overexpressing cells compared to control cells (Figure 7D). Rather, a small decrease in lipogenesis was observed (Figure 7D), which is consistent with a potential role of Ddit4 in regulating fasting responses in adipocytes. Hence, our data on the p53-target Ddit4 presents a valuable example for hypothesis generation from a large-scale data set by suggesting a new role in the fine-tuning of the fasting-response in adipose tissue. However, further investigation is needed to elucidate the exact mechanism by which Ddit4 is enhancing lipolysis in adipocytes, because we show that this is independent form mTORC1 and de-novo lipogenesis.

## Conclusions

In this study, we took a comprehensive view on the fasting process in mice. Our combination of focused and genome-wide approaches reveals several fasting-related findings: (i) We provide a novel view on the immediate and dynamic response to fasting in mice during a 48 hour period. These experiments focus on the timely regulation of liver genes relayed through the Ppara signaling pathway, which manifests in coordinated changes of serum parameters. The observed responses occur very early (between 3 to 6 hours) after onset of fasting and show simultaneous activation of different pathways. (ii) To our knowledge this is the first study focusing on the transcriptome response of white adipose tissue in fasted mice. With our bioinformatic analyses we identify an upregulation of apoptosis-related transcripts as well as a strong enrichment of transcriptional control components in the set of upregulated genes. (iii) Concentrating our analyses on genes regulated in the three tissues mainly responsible for energy homeostasis during fasting (i.e. white adipose tissue, liver, skeletal muscle), the p53 signaling pathway appears to be a common and central regulator of fasting, possibly partly mediating its effect by down-regulation of the Srepb pathway. (iv) Finally, we performed experiments that prove that Ddit4, a p53 target gene upregulated by fasting in all three tissues, is induced by p53 activation and sufficient to increase lipolysis in cultured adipocytes. In conclusion, our transcriptome study of three tissues combined with bioinformatic analyses and mechanistic in vitro experiments, suggests the p53-Ddit4 axis as a novel mechanism in the fine-tuning of fasting common to major metabolic tissues.

## Methods

### Mouse experiments

Experimental animal procedures were in accordance with institutional guidelines and regulations of the University of Pennsylvania. The institutional review board of the University of Pennsylvania reviewed and approved all mouse experiments. Male wild-type C57Bl/6 J mice (Jackson Laboratories) were kept on regular chow (Research Diets) under standard conditions in a 12 hours day/12 hours night cycle. At an age of 10–12 weeks, animals were separated into two groups of 25 mice each. Food was withdrawn from the fasting group at 9 a.m., while the control group had continuous ad libitum access to their diet. Blood glucose was always determined before sacrificing mice. Mice were sacrificed at the beginning of the study (0 hours) and at 3, 6, 12, 24, and 48 hours after food removal. Mice were sacrificed by CO_2_ inhalation and blood was collected by intra-cardiac puncture, serum isolated and stored at −80°C. Liver, epididymal adipose tissue, and skeletal muscle of the thigh were dissected in that order, flash frozen in liquid N_2_ and stored at −80°C until mRNA extraction**.** For western blot of Ddit4 tissues were dissected from six months old, male C57Bl/6 J mice that were fasted overnight or continuously kept on normal chow diet.

### Serum parameters

Blood glucose was measured by standard glucose oxidase glucometer test strips (One Touch Ultra, Fisher). Serum samples were analyzed using commercially available kits for insulin (#90080 Ultra Sensitive Mouse Insulin ELISA Kit, Crystal Chem), NEFAs (#995-34791 and 993–35191, Wako), glycerol (#F6428, Sigma), and β-hydroxybutyrate (#2440-058, Stanbio). Corticosterone levels were determined with a Mouse/Rat Corticosterone ELISA kit (#55-CORMS-E01, Alpco).

### Tissue isolation

Tissues were homogenized using a TissueLyser (Qiagen). mRNA was isolated by RNeasy spin columns and the RNeasy Lipid tissue kit (#74804, Qiagen), if needed. For tissue western blot, tissues were homogenized (Ultra-turax, IKA) in RIPA buffer, incubated 20 min on ice, and centrifuged (1000 g, 10 min, 4°C). Protein phase was isolated and measured with BCA kit (#23227, Pierce).

### qPCR analyses

For qPCR measurements of tissue gene expression, isolated total RNA was reverse transcribed using High Capacity RNA-to-cDNA Master Mix (#4377474) and amplified using TaqMan Universal PCR Master Mix (#4324018) and measured using gene-specific Assays-on-demand (all Applied Biosystems). Amplifications were performed on an ABI Prism 7900HT machine following manufacturer’s protocols. PCR efficiency was calculated from standard curves and the expression of 36b4 (time series measurements) or Gapdh (verification of microarray measurements, Figure 5) was used for normalization. For cell culture experiments SYBR green qPCR was used. Total RNA was isolated with the GeneElute Mammalian Total RNA kit (#RTN70, Sigma). For reverse transcription Qiagen QuantiTect RT kit (#205311) was used. cDNA was then amplified using Sybr QPCR supermix (#11733-038, Life Technologies) on an ABI 7000 sequence detection system. Primers used Ddit4 (Fw: CCTGCGCGTTTGCTCATGCC; Rev: GGCCGCACGGCTCACTGTAT); Cdkn1a (Fw: GTCTGAGCGGCCTGAAGATTC; Rev: TGTTCCGGGCCCACCCGGGG); Sesn2 (Fw: CGCCACTCAGAGAAGGTTCA; Rev: ACGGGGTAGTCAGGTCATGT); Lpin1 (Fw: GTCGTCGAGCAAGACAGATTCC; Rev: ACCAGGATCCCCATTCTTGG); Srebf1 (Fw: AAGCAAATCACTGAAGGACCTGG; Rev: AAAGACAAGGGGCTACTCTGGGAG); Pparg2 (Fw: TGCCTATGAGCACTTCACAAGAAAT; Rev: CGAAGTTGGTGGGCCAGAA); ATGL (Fw: GTCCTTCACCATCCGCTTGTT; Rev: CTCTTGGCCCTCATCACCAG); HSL (Fw: CCATCTCACCTCCCTTGG; Rev: TCCTTCCCGTAGGTCATAGG); Plin1 (Fw: GGTACACTATGTGCCGCTTCC; Rev: CTTTGCGCTCCGCCTCT); 36b4 was used for normalizaiton (Fw: CAACCCAGCTCTGGAGAAAC; Rev: CCAACAGCATATCCCGAATC). Expression values were calculated with an in-house tool [89] employing the AnalyzerMiner algorithm [90].

### Microarray experiments

Frozen samples of white adipose tissue, liver, and skeletal muscle collected at the 24 hour time point were used for microarray experiments. RNA samples were quantified using a NanoDrop (ND-1000). RNA integrity was examined using an Agilent 2100 Bioanalyzer. RNA samples (150 ng) with RNA integrity number >7 were used for target amplification and labeling via the Ambion WT Expression kit (#4411974) and Affymetrix WT Terminal Labeling kit (#900671) following manufacturer’s protocol. Mouse Gene 1.1 ST Array Plates (#901418, Affymetrix) were used for microarray hybridization, wash, stain and scan with GeneTitan hyb-wash-stain kits (#901622, Affymetrix) and a GeneTitan instrument. GeneTitan scanner data were collected with default parameters and further analyzed using Partek Genomics Suite. Data were normalized using default RMA method. A two-way ANOVA model with an interaction term between diet (fed or fasted) and tissue (liver, skeletal muscle and white adipose tissue) was set up. Pairwise comparisons were made between fed and fasted diet for each tissue. The resulting p-values of significance were corrected for multiple testing using Benjamini-Hochberg’s false discovery rate (FDR) method. Genes within 5% FDR and changed at least by 1.3-fold in either direction were called differentially expressed. Data was deposited in NCBI gene expression omnibus (GEO) with the accession number GSE46495.

### Functional annotations and mappings

For DAVID functional annotation, Gene IDs of differentially regulated gene lists (1.3x, FDR5) were submitted to the DAVID website [43,91]. GO_FAT terms and KEGG pathways were considered significantly enriched if the Benjamini-Hochberg corrected p-value was >0.05. For functional clustering only enrichment scores (negative logarithm of geometric mean of p-values of entities in each cluster) larger than three were considered because they contain at least one entity with a significant p-value after multiple testing correction (<0.05). Gene-set enrichment analysis [45] was performed with all genes from the WAT microarrays as “expression data set” and the lists of transcription factor sub-classes, cofactors, and chromatin remodelers from AnimalTFDB [44] as “gene sets database”. The Venn diagram for intersection of genes differentially expressed in all tissues was drawn using BioVenn [92]. MetaCore enrichment analysis matches gene IDs of possible targets for the “common”, “similar” and ”unique” sets (differentially expressed in all 3, 2 out of 3, and only 1 tissue, respectively) with gene IDs in functional ontologies in MetaCore. The probability of a random intersection between a set of IDs the size of target list with ontology entities is estimated in p-value of hypergeometric intersection. Heatmaps for Figure 4 were generated with Genesis [93].

### cDNA cloning

The Ddit4 coding sequence was PCR-amplified with coding sequence-flanking primers from mouse adipose tissue cDNA and cloned into a pMSCV mammalian expression vector (Life Technologies) between XhoI and EcoRI restriction sites using standard procedures. Correct cloning was verified by sequencing of the whole insert.

### Cell culture experiments

C3H10T1/2 cells were maintained in growth medium (High-glucose Dulbecco’s modified Eagle’s medium supplemented with 10% FBS, 2 mM L-glutamine, 100 U/ml penicillin, 100 mg/ml streptomycin (all from Life Technologies)). Two days post-confluent cells were induced to undergo adipogenesis by addition of 1 μM dexamethasone, 500 μM 3-isobutyl-1-methylxanthine, 5 μg/ml insulin (all Sigma), and 1 μM rosiglitazone (Alexis) as described by others [94]. From day 3 on growth medium was only supplemented with 1 μg/ml insulin for 2 days before switching back to normal growth medium. For Nutlin-3 treatments day 7 adipocytes were treated for 6 hours with 10 μM of Nutlin-3 (Sigma) or DMSO as control, before cells were harvested for RNA and protein analysis. For Ddit4 overexpression day 7 adipocytes were detached with a trypsin (0.25%)/collagenase (0.5 mg/ml) mix, washed and resuspended in electroporation buffer R (Neon electroporation kit, #MPK1025, Life Technologies) containing 1 μg of either empty overexpression vector (pMSCV, Life Technologies) or vector with Ddit4 coding sequence at a concentration of 30,000 cells/μl. Electorporation was performed in 10 μl tips with 1400 V/30 ms pulses using a Neon transfection system (Life Technologies). Three electroporation reactions were reseeded in one well of a 12-well plate in growth medium without antibiotics, which was replaced by normal growth medium on the next day for RNA, protein and glycerol measurements. For FFA measurements 2% FFA-free BSA (#K31-002, PAA) containing growth medium without FBS was used. These media were supplemented with 100 nM rapamycin or 1 μM isoproterenol for 1 hour where indicated. After 48 hours, cells were harvested for protein and RNA analysis, and supernatants were collected for glycerol and FFA determination.

### Free fatty acid and glycerol measurements

Collected media of electroporated cells were centrifuged at 12,000 g for 5 min and supernatants were transferred to new tubes. FFA and glycerol contents were measured using commercial kits (from Wako (#NEFA-HR(2)) and Thermo Scientific (#TR22421), respectively) according to the manufacturer’s protocol. Concentrations were derived from standard values/curves and related to the amount of protein in the same well as determined with BCA assay (#23227, Pierce). Finally, values were related to the empty vector control measurements to obtain relative glycerol/FFA release in per cent.

### Lipogenesis assay

C3H10T1/2 adipocytes were incubated overnight with medium (no-glucose DMEM, 10% FBS, 50 μg/mL streptomycin, 50 units/mL penicillin) supplemented with 0.5 g/L glucose and 0.1 μCi D[^14^C(U)]-glucose/ml (ARC). Cells were washed three times with ice-cold PBS before cellular lipids were extracted with hexane/isopropanol (3:2, vol). The incorporated radioactivity in the organic phase was determined by liquid scintillation counting. Counted values were corrected by protein content.

### Western blot analysis

Western blot analysis was performed as we described previously [95] with the following changes: SDS-lysis buffer was supplemented with phosphatase inhibitor (PhosStop, Roche) to reduce changes in phosphorylation states, a 4-12% Bis-Tris gel (Life Technologies) was used and 40 μg protein was loaded per lane. Antibodies used: α-Ddit4 (# 10638-1-AP, ProteinTech Europe), α-S6K1 (#9202, Cell Signaling), α-phospho S6K1 (Thr389) (#9206, Cell Signaling). For tissue western blot 70 μg of protein was loaded. The following antibodies were used to detect loading controls: β-actin (#A5316, Sigma) for WAT and LIV and β-tubulin (#T5201, Sigma) for SM. Detection was performed using ECL prime substrate from GE Healthcare. Before reprobing blots were stripped with Restore WB stripping buffer from Pierce.

### Statistical analyses

To determine statistical significance in time series measurements a 2-way ANOVA was used followed by a Bonferroni posttest to determine significant differences for the single time points. For comparative qPCR measurements upon Ddit4 overexpression and Nutlin-3 treatment a two-tailed, unpaired student’s t-test was used and for FFA, glycerol and lipogenesis measurements a one-sample t-test. A p < 0.05 was considered as statistically significant (*p < 0.05; **p < 0.01; ***p < 0.001). qPCR time series measurements and bar graphs are shown as average ± SEM from independent experiments (sample size as indicated in figure legends). Significance computation in the microarray data was performed using a two-way ANOVA model with a Benjamini-Hochberg’s false discovery rate (FDR) to correct for multiple testing. In this study, transcripts with an FDR5 and >1.3-fold difference were considered as differentially expressed between fasted and fed groups. For DAVID analyses GO terms and KEGG pathways were considered as significantly enriched if the Benjamini-Hochberg’s corrected p-value was <0.05 [43]. Significant enrichment of gene lists in transcription factors, cofactors, and chromatin remodelers was tested with a Chi-square test with Yate’s correction (http://graphpad.com/quickcalcs/contingency1.cfm).

## Abbreviations

WAT: Epididymal white adipose tissue; LIV: Liver; SM: Skeletal muscle; NEFA: Non-esterified fatty acids; GNG: Gluconeogenesis; GO: Gene Ontology; KEGG: Kyoto Encyclopedia of Genes and Genomes; Ppara: Peroxisome proliferator activated receptor alpha; Ppargc1a: Peroxisome proliferative activated receptor, gamma, coactivator 1 alpha; Fgf21: Fibroblast growth factor 21; Pck1: Phosphoenolpyruvate carboxykinase 1; G6pc: Glucose-6-phosphatase, catalytic; Hmgcs2: 3-hydroxy-3-methylglutaryl-Coenzyme A synthase 2; Gyk: Glycerol kinase; Pcx: Pyruvate carboxylase; Irf4: interferon regulatory factor 4; Zim1: Zinc finger, imprinted 1; Peg3: Paternally expressed 3; Cdkn1a: Cyclin-dependent kinase inhibitor 1A; Lpin1: Lipin1; Sesn1/2: Sestrin 1/2; Srebf1/2: Sterol regulatory element binding transcription factor 1/2; Acss2: Acyl-CoA synthetase short-chain family member 2; Acaca: Acetyl-Coenzyme A carboxylase alpha; Fasn: Fatty acid synthase; Scd1/2: Stearoyl-Coenzyme A desaturase 1/2; Ddit4: DNA-damage-inducible transcript 4; Cebpd: CCAAT/enhancer binding protein, delta; Per1: Period homolog 1; Pparg2: Peroxisome proliferator activated receptor gamma 2; ATGL: Adipose triglyceride lipase; HSL: Hormone-sensitive lipase; Plin1: Perilipin 1; S6K1: Ribosomal protein S6 kinase, polypeptide 1; mTORC1: Mechanistic target of rapamycin complex 1; Dex: Dexamethasone; Rapa: Rapamycin.

## Competing interests

The authors declare that they have no competing interests.

## Authors’ contributions

AP conceptualized and wrote the manuscript, performed the cell culture experiments, and analyzed the microarray and qPCR data; MS designed and performed mouse experiments, carried out gene expression studies and metabolite measurements, and contributed to the writing of the manuscript; ERB performed mouse experiments and carried out gene expression studies and metabolite measurements; ARP performed mouse experiments; HJP performed the lipogenesis assay; MAL designed experiments, interpreted data, and contributed to the writing of the manuscript; FC and DB conceived and performed the microarray experiments and qPCR validations; SR analyzed data; JGB designed experiments and assisted in writing the manuscript. All authors read and approved the final manuscript.

## Supplementary Material

Additional file 1 Lists of probes expressed more than 1.3-fold with an FDR5 for each of the three tissues; p-value given is corrected for multiple testing. Click here for file

Additional file 2 Detailed results of the functional clustering by DAVID including terms/pathways contributing to each cluster; Lists of genes up- and downregulated by 24 hours of fasting in WAT where submitted separately and resulting clusters are listed in separate tabs. Click here for file

Additional file 3 Annotation of genes regulated in fasting WAT according to AnimalTFDB; Results of GSEA analysis testing for enrichment in AnimalTFDB categories (sub-categories of transcription factors (TF), cofactor (CoF), chromatin remodelers (ChrRem)); Manual mapping of differentially expressed WAT genes in the AnimalTFDB categories TF, CoF, and ChrRem; GSEA results for gene sets enriched in up- or downregulated genes (by fasting in WAT) using c2 and c5 (MSigDB) modules as gene sets. Click here for file

Additional file 4 Metacore analysis focusing on networks overrepresented in the common list reveals the p53 node as the second highest scoring network hub. Click here for file

Additional file 5 Metacore pathway analysis on Affymetrix-probes regulated commonly by fasting in WAT, LIV, and SM yields Srebp-regulated pathways as 2 top-scoring entities. Click here for file

Additional file 6 DAVID mapping to GO_BP_FAT of up- or downregulated genes for WAT, LIV, and SM; only significant terms are depicted. Click here for file
