# Supplementary material for: Metabolite and transcriptome analysis during fasting suggest a role for the p53-Ddit4 axis in major metabolic tissues
Source: BMC Genomics. 2013 Nov 5;14:758. doi: 10.1186/1471-2164-14-758 (PMC3907060; doi:10.1186/1471-2164-14-758)
Supplement: Additional file 5 — Metacore pathway analysis on Affymetrix-probes regulated commonly by fasting in WAT, LIV, and SM yields Srebp-regulated pathways as 2 top-scoring entities. [file 1471-2164-14-758-S5.pdf]

Compare Experiments Workflow 1.0 Data Analysis Report

Server: *portal.genego.com*
Date: *2011-08-23*
Name: *University of Pennsylvania - John Tobias named seat - LPA 1/27/11*
Login: *upenn13*

Experiments

1.		FAT_fastedvsfed_input_byFDR
2.		SM_fastedvsfed_input_byFDR
3.		liver_fastedvsfed_input_byFDR

Figure 1. The experiments uploaded for comparative analysis

Contact: Julie Bryant GeneGO, Inc. 169 Saxony Rd., #104, Encinitas CA, 92024
Phone: (858) 756 7996 E-mail: julie@genego.com

Table of content:

- Experiments comparison
- Enrichment analysis
  - GeneGo Pathway Maps
    - Top maps (sorted by common)
      - 1. Map : Regulation of lipid metabolism_Regulation of fatty acid synthase activity in hepatocytes
      - 2. Map : Regulation of lipid metabolism_Regulation of lipid metabolism via LXR, NF-Y and SREBP
      - 3. Map : Development_Role of IL-8 in angiogenesis
      - 4. Map : Regulation of metabolism_Bile acids regulation of glucose and lipid metabolism via FXR
      - 5. Map : Cell cycle_Regulation of G1/S transition (part 1)

Contact: Julie Bryant GeneGO, Inc. 169 Saxony Rd., #104, Encinitas CA, 92024
Phone: (858) 756 7996 E-mail: julie@genego.com

Experiments comparison

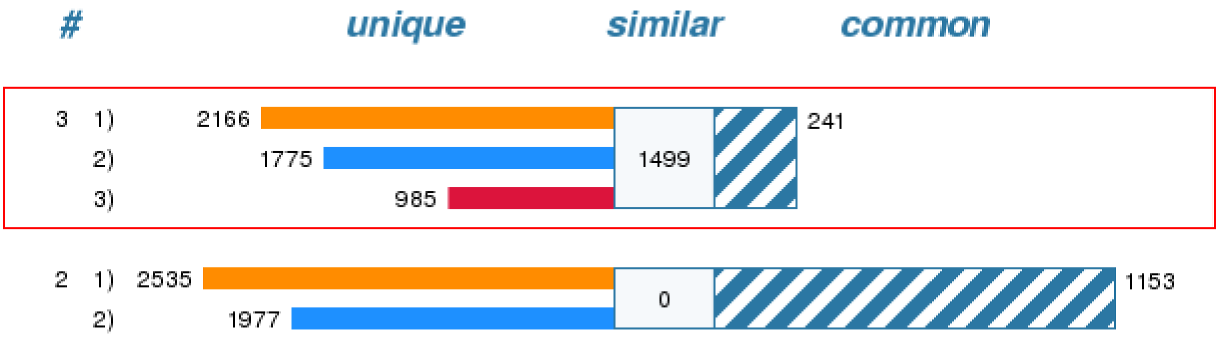

Figure 2. The gene content is aligned between all uploaded experiments. The intersection set of experiments is defined as 'common' and marked as a blue/white striped bar. The unique genes for the experiments are marked as colored bars. The genes from the 'similar' set are present in all but one (any) file. The parameters for comparison are set as above.

Contact: Julie Bryant GeneGO, Inc. 169 Saxony Rd., #104, Encinitas CA, 92024
Phone: (858) 756 7996 E-mail: julie@genego.com

Enrichment analysis

Enrichment analysis consists of matching gene IDs of possible targets for the "common", "similar" and "unique" sets with gene IDs in functional ontologies in MetaCore. The probability of a random intersection between a set of IDs the size of target list with ontology entities is estimated in p-value of hypergeometric intersection. The lower p-value means higher relevance of the entity to the dataset, which shows in higher rating for the entity.

Ontologies available for EA in Compare Experiments Workflow:

Contact: Julie Bryant GeneGO, Inc. 169 Saxony Rd., #104, Encinitas CA, 92024
Phone: (858) 756 7996 E-mail: julie@genego.com

GeneGo Pathway Maps

Canonical pathway maps represent a set of about 650 signaling and metabolic maps covering human biology (signaling and metabolism) in a comprehensive way. All maps are drawn from scratch by GeneGo annotators and manually curated & edited. Experimental data is visualized on the maps as blue (for downregulation) and red (upregulation) histograms. The height of the histogram corresponds to the relative expression value for a particular gene/protein.

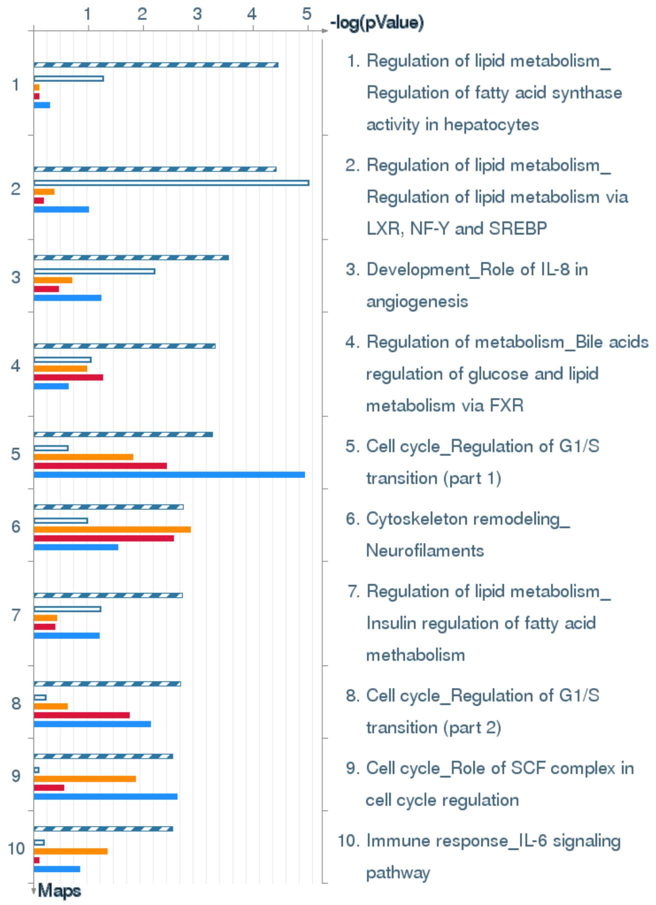

Figure 3. GeneGo Pathway Maps. Sorting is done for the 'common' set.

Contact: Julie Bryant GeneGO, Inc. 169 Saxony Rd., #104, Encinitas CA, 92024
Phone: (858) 756 7996 E-mail: julie@genego.com

Top maps (sorted by common)

1. Map : Regulation of lipid metabolism_Regulation of fatty acid synthase activity in hepatocytes

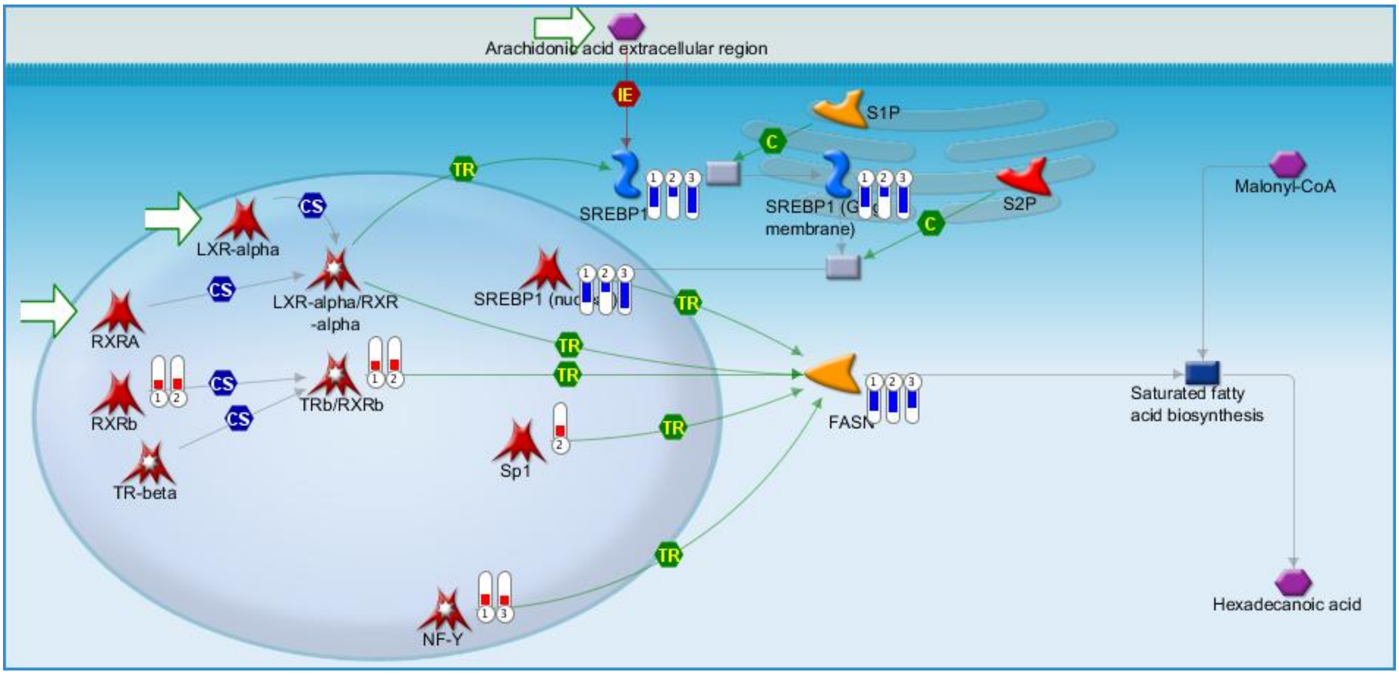

Figure 4. The top scored map (map with the the lowest p-value) based on the enrichment distribution sorted by 'common' set. Experimental data from all files is linked to and visualized on the maps as thermometer-like figures. Up-ward thermometers have red color and indicate up-regulated signals and down-ward (blue) ones indicate down-regulated expression levels of the genes.

Contact: Julie Bryant GeneGO, Inc. 169 Saxony Rd., #104, Encinitas CA, 92024
Phone: (858) 756 7996 E-mail: julie@genego.com

2. Map : Regulation of lipid metabolism_Regulation of lipid metabolism via LXR, NF-Y and SREBP

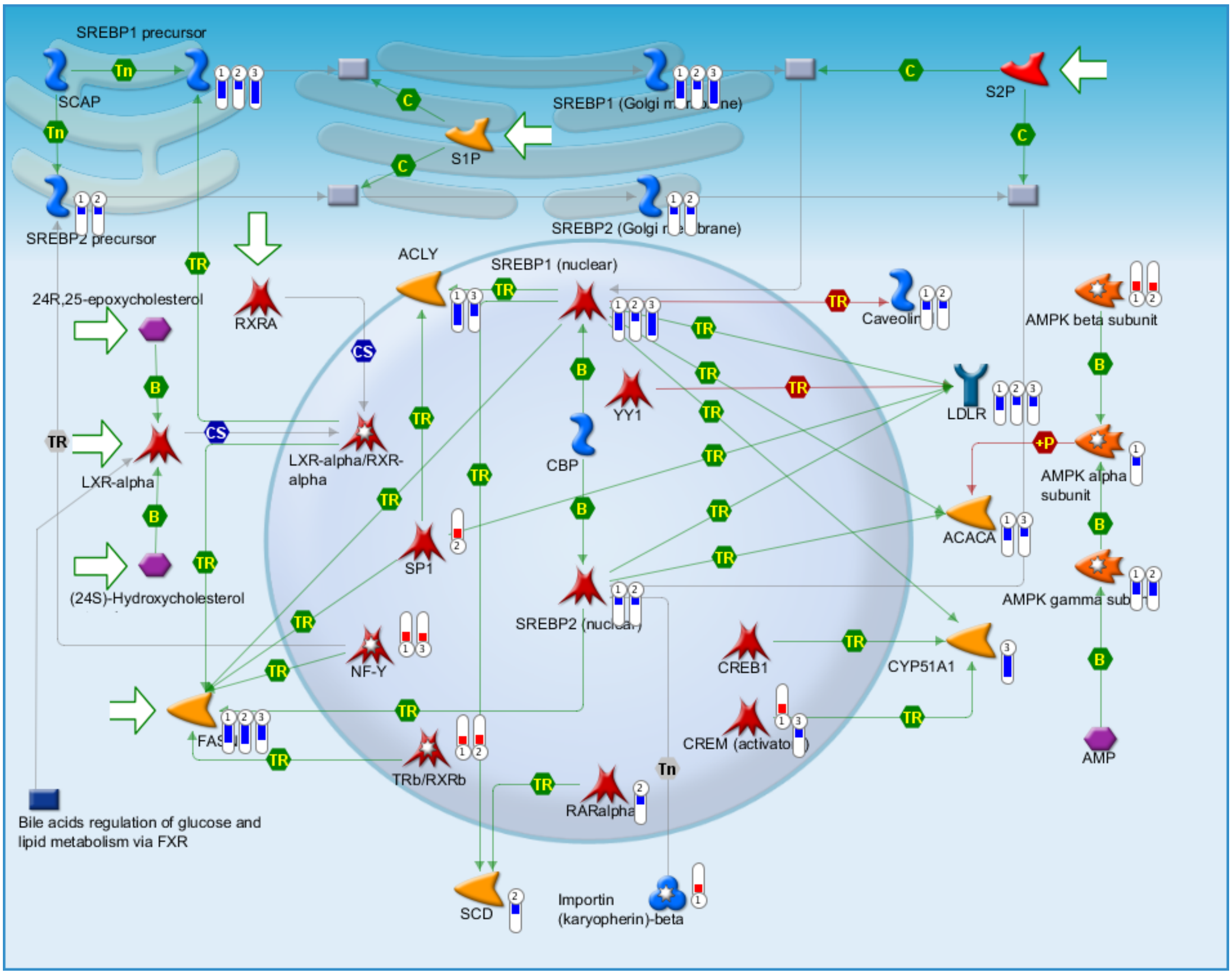

Figure 5. The second scored map (map with the second lowest p-value) based on the enrichment distribution sorted by 'common' set. Experimental data from all files is linked to and visualized on the maps as thermometer-like figures. Up-ward thermometers have red color and indicate up-regulated signals and down-ward (blue) ones indicate down-regulated expression levels of the genes.

Contact: Julie Bryant GeneGO, Inc. 169 Saxony Rd., #104, Encinitas CA, 92024
Phone: (858) 756 7996 E-mail: julie@genego.com

3. Map : Development_Role of IL-8 in angiogenesis

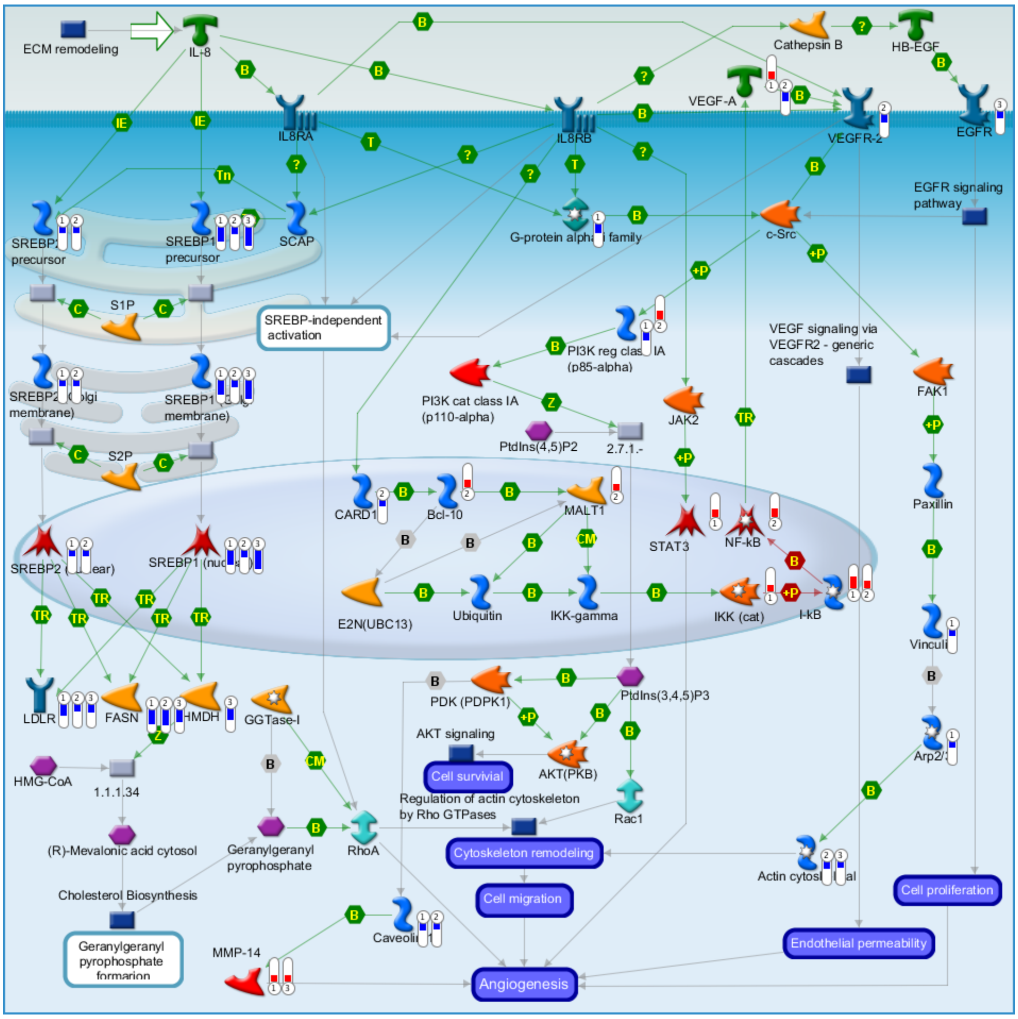

Figure 6. The third scored map (map with the third lowest p-value) based on the enrichment distribution sorted by 'common' set. Experimental data from all files is linked to and visualized on the maps as thermometer-like figures. Up-ward thermometers have red color and indicate up-regulated signals and down-ward (blue) ones indicate down-regulated expression levels of the genes.

Contact: Julie Bryant GeneGO, Inc. 169 Saxony Rd., #104, Encinitas CA, 92024
Phone: (858) 756 7996 E-mail: julie@genego.com

4. Map : Regulation of metabolism_Bile acids regulation of glucose and lipid metabolism via FXR

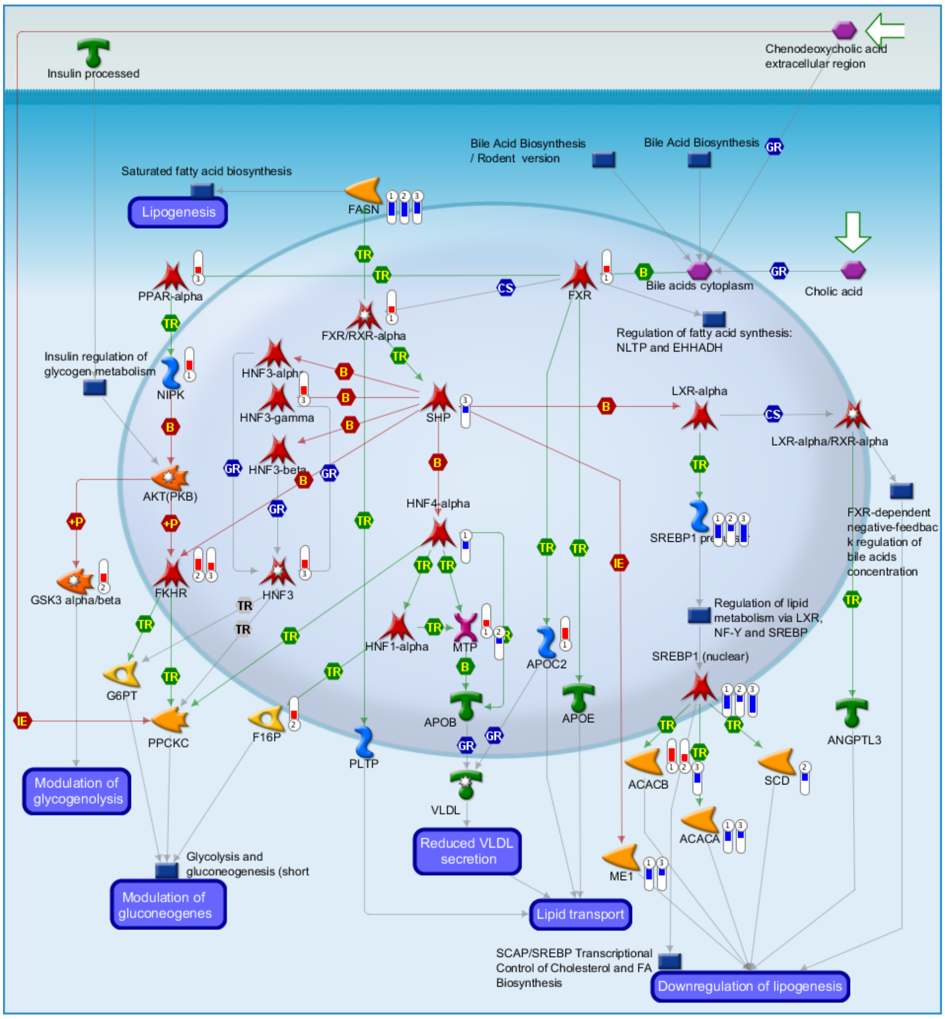

Figure 7. The fourth scored map (map with the fourth lowest p-value) based on the enrichment distribution sorted by 'common' set. Experimental data from all files is linked to and visualized on the maps as thermometer-like figures. Up-ward thermometers have red color and indicate up-regulated signals and down-ward (blue) ones indicate down-regulated expression levels of the genes.

Contact: Julie Bryant GeneGO, Inc. 169 Saxony Rd., #104, Encinitas CA, 92024
Phone: (858) 756 7996 E-mail: julie@genego.com

5. Map : Cell cycle_Regulation of G1/S transition (part 1)

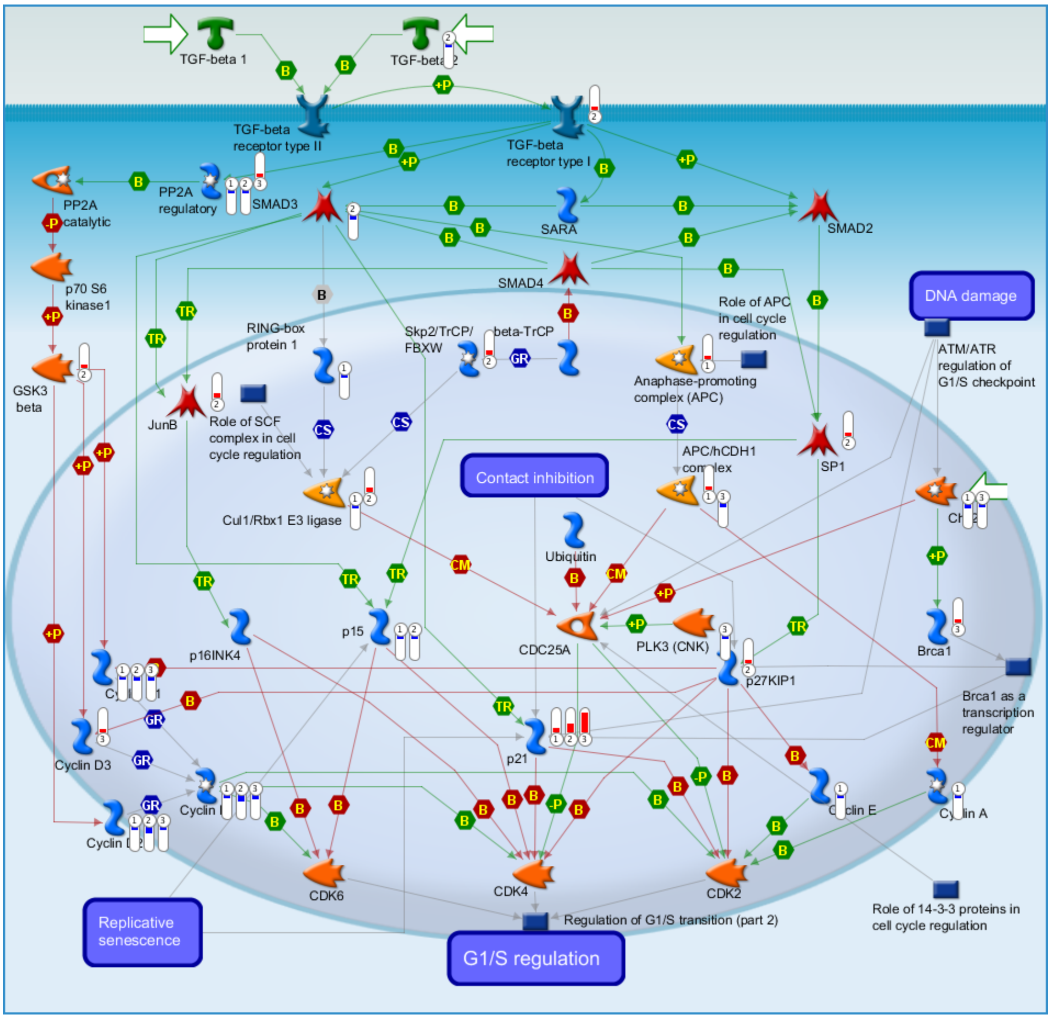

Figure 8. The fifth scored map (map with the fifth lowest p-value) based on the enrichment distribution sorted by 'common' set. Experimental data from all files is linked to and visualized on the maps as thermometer-like figures. Up-ward thermometers have red color and indicate up-regulated signals and down-ward (blue) ones indicate down-regulated expression levels of the genes.

Contact: Julie Bryant GeneGO, Inc. 169 Saxony Rd., #104, Encinitas CA, 92024
Phone: (858) 756 7996 E-mail: julie@genego.com
