# Supplementary material for: Metabolite and transcriptome analysis during fasting suggest a role for the p53-Ddit4 axis in major metabolic tissues
Source: BMC Genomics. 2013 Nov 5;14:758. doi: 10.1186/1471-2164-14-758 (PMC3907060; doi:10.1186/1471-2164-14-758)
Supplement: Supplementary file 6 [file 1471-2164-14-758-S6.docx]

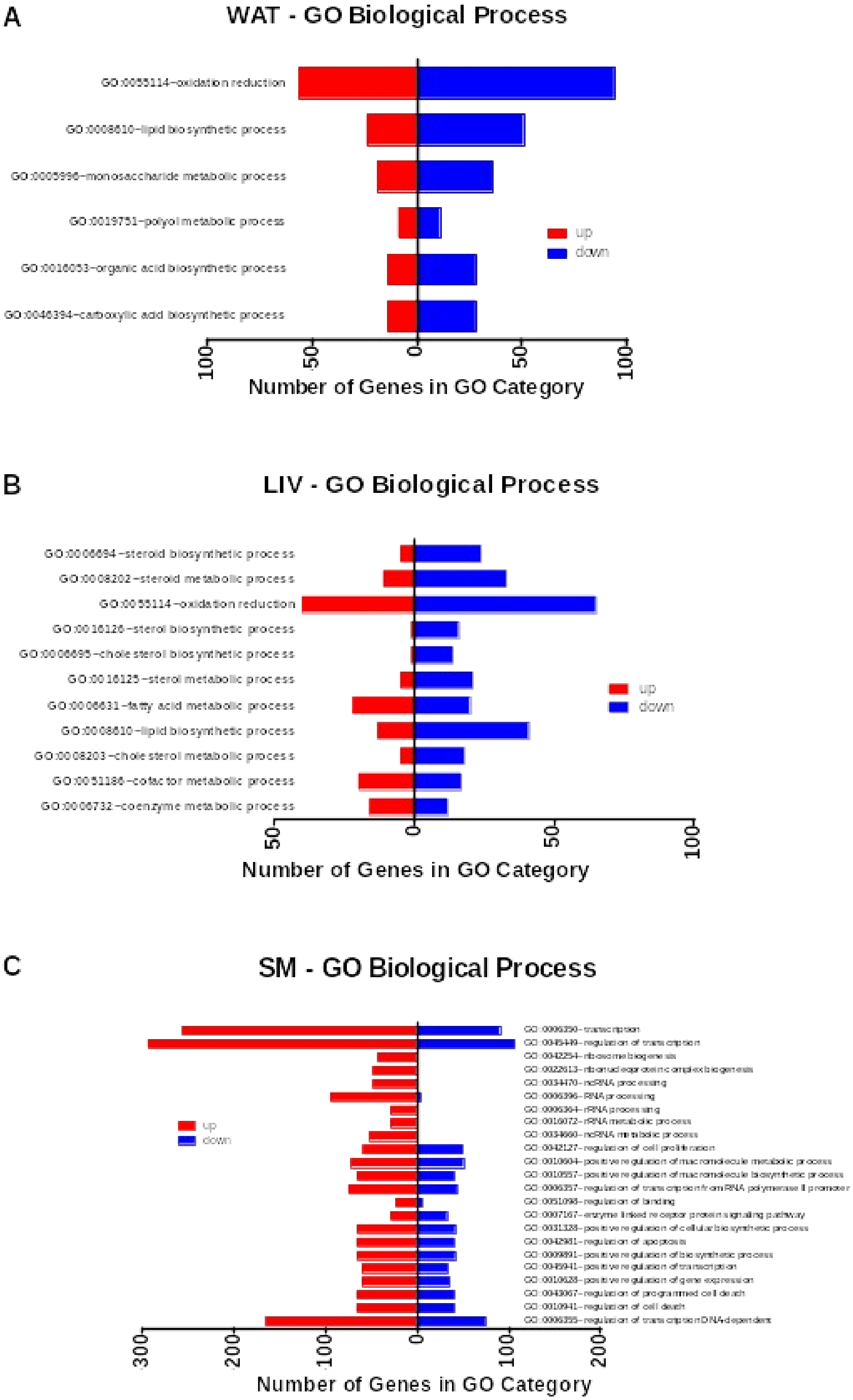
A
WAT - GO Biological Process
GO:0055114~oxidation reduction
GO:0008610~lipid biosynthetic process
GO:0005996~monosaccharide metabolic process
GO:0019751~polyol metabolic process
GO:0016053~organic acid biosynthetic process
GO:0046394~carboxylic acid biosynthetic process
up
down
100
50
0
50
100
Number of Genes in GO Category
B
LIV - GO Biological Process
GO:0006694~steroid biosynthetic process
GO:0008202~steroid metabolic process
GO:0055114~oxidation reduction
GO:0016126~sterol biosynthetic process
GO:0006695~cholesterol biosynthetic process
GO:0016125~sterol metabolic process
GO:0006631~fatty acid metabolic process
GO:0008610~lipid biosynthetic process
GO:0008203~cholesterol metabolic process
GO:0051186~cofactor metabolic process
GO:0006732~coenzyme metabolic process
up
down
50
0
50
100
Number of Genes in GO Category
C
SM - GO Biological Process
GO:0006350~transcription
GO:0045449~regulation of transcription
GO:0042254~ribosome biogenesis
GO:0022613~ribonucleoprotein complex biogenesis
GO:0034470~ncRNA processing
GO:0006396~RNA processing
GO:0006364~rRNA processing
GO:0016072~rRNA metabolic process
GO:0034660~ncRNA metabolic process
GO:0042127~regulation of cell proliferation
GO:0010604~positive regulation of macromolecule metabolic process
GO:0010557~positive regulation of macromolecule biosynthetic process
GO:0006357~regulation of transcription from RNA polymerase II promoter
GO:0051098~regulation of binding
GO:0007167~enzyme linked receptor protein signaling pathway
GO:0031328~positive regulation of cellular biosynthetic process
GO:0042981~regulation of apoptosis
GO:0009891~positive regulation of biosynthetic process
GO:0045941~positive regulation of transcription
GO:0010628~positive regulation of gene expression
GO:0043067~regulation of programmed cell death
GO:0010941~regulation of cell death
GO:0006355~regulation of transcription DNA-dependent
up
down
300
200
100
0
100
200
Number of Genes in GO Category
